# metamicrobiomeR: an R package for analysis of microbiome relative abundance data using zero-inflated beta GAMLSS and meta-analysis across studies using random effects models

**DOI:** 10.1186/s12859-019-2744-2

**Published:** 2019-04-16

**Authors:** Nhan Thi Ho, Fan Li, Shuang Wang, Louise Kuhn

**Affiliations:** 1 Gertrude H. Sergievsky Center, Columbia University, New York City, NY USA; 2 Institute of Applied Sciences and Regenerative Medicine, Vinmec Healthcare System, 458 Minh Khai, Hai Ba Trung, Ha Noi, Vietnam; 3 Department of Pediatrics, University of California, Los Angeles, CA USA; 4 Department of Biostatistics, Mailman School of Public Health, Columbia University, New York City, NY USA

**Keywords:** Microbiome, Relative abundance, GAMLSS, Zero-inflated beta, Meta-analysis, Random effect, Pooling estimates, Infant, Gender

## Abstract

**Background:**

The rapid growth of high-throughput sequencing-based microbiome profiling has yielded tremendous insights into human health and physiology. Data generated from high-throughput sequencing of 16S rRNA gene amplicons are often preprocessed into composition or relative abundance. However, reproducibility has been lacking due to the myriad of different experimental and computational approaches taken in these studies. Microbiome studies may report varying results on the same topic, therefore, meta-analyses examining different microbiome studies to provide consistent and robust results are important. So far, there is still a lack of implemented methods to properly examine differential relative abundances of microbial taxonomies and to perform meta-analysis examining the heterogeneity and overall effects across microbiome studies.

**Results:**

We developed an R package ‘*metamicrobiomeR’* that applies Generalized Additive Models for Location, Scale and Shape (GAMLSS) with a zero-inflated beta (BEZI) family (GAMLSS-BEZI) for analysis of microbiome relative abundance datasets. Both simulation studies and application to real microbiome data demonstrate that GAMLSS-BEZI well performs in testing differential relative abundances of microbial taxonomies. Importantly, the estimates from GAMLSS-BEZI are log (odds ratio) of relative abundances between comparison groups and thus are analogous between microbiome studies. As such, we also apply random effects meta-analysis models to pool estimates and their standard errors across microbiome studies. We demonstrate the meta-analysis examples and highlight the utility of our package on four studies comparing gut microbiomes between male and female infants in the first six months of life.

**Conclusions:**

GAMLSS-BEZI allows proper examination of microbiome relative abundance data. Random effects meta-analysis models can be directly applied to pool comparable estimates and their standard errors to evaluate the overall effects and heterogeneity across microbiome studies. The examples and workflow using our ‘*metamicrobiomeR’* package are reproducible and applicable for the analyses and meta-analyses of other microbiome studies.

**Electronic supplementary material:**

The online version of this article (10.1186/s12859-019-2744-2) contains supplementary material, which is available to authorized users.

## Background

The rapid growth of high-throughput sequencing-based microbiome profiling has yielded tremendous insights into human health and physiology. However, interpretation of microbiome studies have been hampered by a lack of reproducibility in part due to the variety of different study designs, experimental approaches, and computational methods used [1, 2]. Microbiome studies may report varying results on the same topic. Therefore, meta-analyses examining different microbiome studies are critical to provide consistent robust results. Although many methods for microbiome differential abundance analysis have been proposed, methods for meta-analysis remain underdeveloped. Meta-analysis studies pooling individual sample data across studies for pooled analysis of all samples or processing of all samples together followed by analysis of each study separately have revealed some consistent microbial signatures in certain conditions such as inflammatory bowel disease (IBD) and obesity [3–9]. Software has been developed for the analysis and meta-analysis of microbiome data [10]. However, these studies do not explicitly model microbiome relative abundance data using an appropriate statistical method and do not examine between-group comparison overall pooled effects in the meta-analysis.

Data generated from high-throughput sequencing of 16S rRNA gene amplicons are often preprocessed into relative abundance. Microbiome relative abundances are compositional data which range from zero to one and are generally zero-inflated. To test for differences in relative abundance of microbial taxonomies between groups, methods such as bootstrapped non-parametric t-tests or Wilcoxon tests (not suitable for longitudinal data and covariate adjustment) [11–13] and linear or linear mixed effect models (LM) [14, 15] (suitable for longitudinal data and covariate adjustment) have been widely used. However, these methods do not address the actual distribution of the microbial taxonomy relative abundance data, which resemble a zero-inflated beta distribution. Transformations (e.g. arcsin square root) of relative abundance data to make it resemble continuous data to use in LM has been proposed by Morgan et al. (implemented in MaAsLin software) [16] and has been widely used to test for differential relative abundances [17–20]. However, this adjustment does not address the inflation of zero values in microbiome relative abundance data.

Various methods for the analysis of differential abundance based have been proposed. For example, the zero-inflated Gaussian distribution mixture model regards zero values as under-sampling and account for it by posterior probability estimates and fit counts after accounting for under-sampling by a log-normal distribution [21]. The Ratio Approach for Identifying Differential Abundance (RAIDA) method uses the ratio between the counts of features in each sample to address possible problems associated with counts on different scales within and between conditions and accounts for ratios with zeros using a modified zero-inflated lognormal (ZIL) model treating the zeros as under-sampling [22]. Other methods adapted from the RNA-seq field that account for zero inflation and utilize Poisson or negative binomial models have shown some promise in differential abundance testing of microbiome datasets [23, 24]. These aforementioned methods treat the dispersion as a nuisance parameter and do not allow the dispersion to depend on covariates. Recently, Chen et al. proposed an omnibus test based on a zero-inflated negative model (ZINB) that allows differential analysis not only for feature abundance but also prevalence and dispersion [25]. However, the downside of these count-based methods is the increased complexity due to modeling the counts.

Here, we developed an R package *‘metamicrobiomeR’* that applies Generalized Additive Models for Location, Scale and Shape (GAMLSS) with a zero-inflated beta (BEZI) family (GAMLSS-BEZI) for the analysis of microbial taxonomy relative abundance data. GAMLSS is a general framework for fitting regression type models in which the response variable can be any distribution [26]. With BEZI family, this model allows direct and proper examination of microbiome relative abundance data, which resemble a zero-inflated beta distribution. In principle, this model is similar to the two-part mixed effect model proposed by Chen et al. [27] in that the presence/absence of the taxon in the samples is modeled with a logistic component and the non-zero abundance of the taxon is modeled with a Beta component. Both logistic and beta components allow covariate adjustment and address longitudinal correlations with subject-specific random effects. The GAMLSS-BEZI is based on the broadly applicable established GAMLSS framework that can be flexibly implemented and applied to different types of data and study designs (e.g. cross-sectional and longitudinal). This is especially useful for later meta-analysis across different studies. The performance of GAMLSS-BEZI was evaluated using simulation studies and real microbiome data. Importantly, the estimates (regression coefficients) from GAMLSS-BEZI are log (odds ratio) of being in the case group (as compared to be in the control group) with changes in relative abundance of a specific bacterial taxon and thus are analogous across microbiome studies and can be directly combined using standard meta-analysis approaches. As such, we apply random effects meta-analysis models to pool the estimates and standard errors as part of the ‘*metamicrobiomeR*’ package. This approach allows examination of study-specific effects, heterogeneity between studies, and the overall pooled effects across studies. Finally, we provide examples and sample workflows for both components of the ‘*metamicrobiomeR*’ package. Specifically, we use GAMLSS-BEZI to compare relative abundances of the gut microbial taxonomies of male versus female infants’ ≤6 months of age while adjusting for feeding status and infant age at time of sample collection and demonstrate the application of the random effects meta-analysis component on four studies of the infant gut microbiome.

## Implementation

### GAMLSS-BEZI for the analysis of bacterial taxa relative abundance and bacterial predicted functional pathway relative abundance data

Relative abundances of bacterial taxa at various taxonomic levels (from phylum to genus or species) are obtained via the “*summarize_taxa.py*” script in QIIME1 [13]. Bacterial functional pathway abundances (e.g. Kyoto Encyclopedia of Genes and Genomes (KEGG) pathway level 1 to 3) are obtained from metagenome prediction analysis using PICRUSt [28]. In the *taxa.compare* function, all bacterial taxa or pathway data are first filtered to retain features with mean relative abundance ≥ relative abundance threshold (e.g. ≥0.005%) and with prevalence ≥ prevalence threshold (e.g. present in ≥5% of the total number of samples). This pre-filtering step has been shown to improve performance of various differential abundance detection strategies [29]. A filtered data matrix is then modeled by GAMLSS-BEZI and (μ) logit link and other default options using the R package ‘gamlss’ version 5.0–5 [26]. For longitudinal data, subject-specific random effects can be added to the model. We only include subject random intercepts as in practice this is often sufficient to address the longitudinal correlations [30]. However, it is possible to extend the model to include random slopes depending on the specific research content. For performance evaluation, LM and LM with arcsin squareroot transformation (LMAS) were also implemented in the function *taxa.compare*. In addition, we also implemented different approaches to deal with compositional effects including Centered Log Ratio (CLR) transformation [31] with various zero-replacement options [32] and Geometric Mean of Pairwise Ratios (GMPR) normalization [33]. Multiple testing adjustment can be done using different methods (False Discovery Rate (FDR) control by default). Below is an example call of the *taxa.compare* function:

*taxa.compare (taxtab = taxtab, propmed.rel = “gamlss”, transform = “none”, comvar = “gender”, adjustvar = c(“age.sample”,*“feeding”*),longitudinal = “yes”, percent.filter = 0.05, relabund.filter = 0.00005, p.adjust.method = “fdr”).*

For subsequent meta-analysis, the output from *taxa.compare* comprises matrices containing coefficients, standard errors, *p*-values and multiple testing adjusted p-values of all covariates in the models for each bacterial taxon or pathway.

### Meta-analysis across studies using random effects models

The adjusted regression coefficient estimates from GAMLSS-BEZI are log (odds ratio) of being in the case group (as compared to be in the control group) with changes in relative abundances of a specific bacterial taxa or a pathway and thus are analogous across microbiome studies. Therefore, standard meta-analysis approaches can be directly applied. In the *meta.taxa* function, random effects meta-analysis models pooling adjusted estimates and standard errors with inverse variance weighting and the DerSimonian–Laird estimator for between-study variance are implemented to estimate the overall effects, corresponding 95% confidence intervals (CIs) and heterogeneity across studies. A fixed effect meta-analysis model is also implemented for comparison. Meta-analysis is performed only for taxa or pathways observed in ≥ a specified percentage threshold (e.g. 50%) of the total number of included studies. An example call to *meta.taxa* using the output data matrices combined from multiple calls to the *taxa.compare* function is shown below:

*meta.taxa (taxcomdat = combined.taxa.compare.output, summary.measure = “RR”, pool.var = “id”, studylab = “study”, backtransform = FALSE, percent.meta = 0.5, p.adjust.method = “fdr”).*

The output from *meta.taxa* consists of pooled estimates, standard errors, 95% CI, pooled *p*-values and multiple testing adjusted pooled p-values of all covariates for each bacterial taxon or pathway. The *metatab.show* function displays the meta-analysis outputs from *meta.taxa* as table, heatmap, forest plot or combined dataset to be used by the *meta.niceplot* function to generate nicer looking integrated heatmap-forest plot.

All implemented functions in the ‘*metamicrobiomeR’* package are summarized and illustrated in Additional file 1.

## Results and discussion

### Performance of GAMLSS-BEZI: simulation studies

Simulation studies were performed to evaluate type I error and power of GAMLSS-BEZI for testing differential relative abundances of microbial taxonomies as compared to linear/linear mixed models with arcsin squareroot transformation (LMAS) (implemented in MaAsLin software [16]). LMAS was chosen for comparison with GAMLSS-BEZI because it is a commonly used approach for microbiome differential relative abundance testing and similarly to GAMLSS-BEZI, it allows covariate adjustment and can be used for longitudinal or non-longitudinal data. Simulations of zero-inflated beta distribution of microbiome relative abundance data were based on the R package “gamlss.dist” version 5.0–3.

In brief, beta distribution (denoted as *Beta*(*μ*, *ϕ*)) has a density function:

1 $$ f\left(y;\mu, \phi \right)=\frac{\ \boldsymbol{\Gamma} \left(\phi \right)}{\boldsymbol{\Gamma} \left(\mu \phi \right)\boldsymbol{\Gamma} \left(\left(\mathbf{1}-\mu \right)\phi \right)}{y}^{\mu \phi -1}{\left(1-y\right)}^{\left(1-\mu \right)\phi -1},y\in \left(0,1\right) $$

where 0 ≤ *μ* ≤ 1, *ϕ* > 0 and **Γ** (.) is the gamma function. If *y*~*Beta*(*μ*, *ϕ*), then *E*(*y*) = *μ* and *Var*(*y*) = *μ*(1 − *μ*)/(*ϕ* + 1), in which the variance of the dependent variable is defined as a function of the distribution mean *μ* and the precision parameter *ϕ* [34].

Zero-inflated beta distribution is a mixture of beta distribution and a degenerate distribution in a known value c = 0. A parameter α is added to the beta distribution to account for the probability of observations at zero producing a mixture density [34]:

2 $$ f\left(y;\alpha, \mu, \phi \right)=\left\{\begin{array}{c}\alpha, \kern6.5em if\ y=0,\\ {}\left(1-\alpha \right)f\left(y;\mu, \phi \right),\kern0.75em if\ y\in \left(0,1\right),\end{array}\right. $$

#### Type I error

We considered three sample sizes mimicking case-control microbiome studies with small (number of controls [n_1_] = number of cases [n_2_] = 10), medium (n_1_ = n_2_ = 100) and large (n_1_ = n_2_ = 500) scales. For each sample size, relative abundances of a bacterial species were simulated with the same parameters of a zero-inflated beta distribution for case and control groups (μ_1_ = μ_2_ = 0.5, α_1_ = α_2_ = 0.5, ϕ_1_= ϕ_2_ = 5). The simulation was repeated 1000 times. Type I error was calculated for three different alpha levels of 0.01, 0.05 and 0.1. Type I error of GAMLSS-BEZI or LMAS was defined as the proportion of simulations with *p*-values of GAMLSS-BEZI or LMAS less than the corresponding alpha level over 1000 simulations for each sample size. We noted that Type I errors were well controlled in both GAMLSS-BEZI and LMAS (Table 1).

**Table 1:** Type I error of GAMLSS-BEZI and LMAS

Sample size	GAMLSS-BEZI	LMAS	GAMLSS-BEZI	LMAS	GAMLSS-BEZI	LMAS
	Alpha level = 0.01		Alpha level = 0.05		Alpha level = 0.1	
10	0.014	0.012	0.061	0.050	0.114	0.099
100	0.010	0.010	0.051	0.050	0.103	0.098
500	0.010	0.011	0.052	0.052	0.104	0.103

*GAMLSS-BEZI* Generalized Additive Models for Location, Scale and Shape (GAMLSS) with a zero inflated beta (BEZI) family, *LMAS* linear model with arcsin square root transformation (implemented in the software MaAsLin)

### Receiver operating characteristic (ROC) curve and power

We then evaluated the performance of GAMLSS-BEZI vs. LMAS for identifying bacterial species with differential relative abundance between cases and controls. Two types of simulations were performed. First, relative abundances of 800 bacterial species were simulated in which 400 species had no difference between control and case groups (the same parameters of zero-inflated beta distribution for control and case groups: μ_1_ = μ_2_ = Uniform [0.0005,0.3], α_1_ = α_2_ = Uniform [0.1,0.9], ϕ_1_= ϕ_2_ = 5) and 400 species with a true difference between control and case groups. Specifically, four settings for the 400 species with true differences between control and case groups were considered with 100 species for each setting:

1. μ_1_ = Uniform [0.0005,0.3] vs. μ_2_ = μ_1_ + 0.1
2. μ_1_ = Uniform [0.0005,0.3] vs. μ_2_ = μ_1_ + 0.2
3. μ_1_ = Uniform [0.0005,0.3] vs. μ_2_ = μ_1_ + 0.3
4. μ_1_ = Uniform [0.0005,0.3] vs. μ_2_ = μ_1_ + 0.4

Other parameters (α, ϕ) were set the same for control and case groups (α_1_ = α_2_ = Uniform [0.1,0.9], ϕ_1_= ϕ_2_ = 5). A sample size of *n* = 100 for both case and control groups was used.

**Fig. 1:**
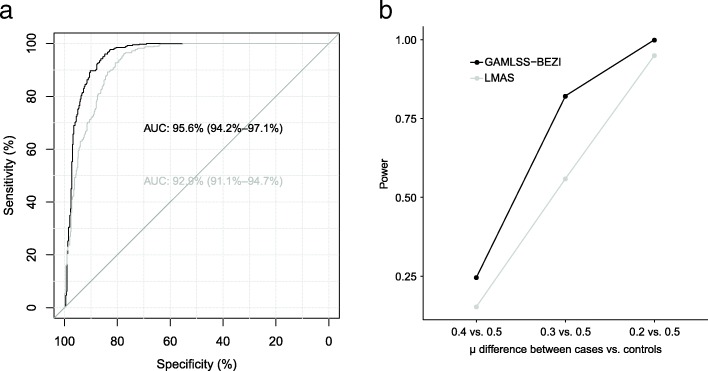
ROC curve and power of GAMLSS-BEZI vs. LMAS. **a**. ROC curve of GAMLSS-BEZI and LMAS for identifying species with differential abundance between case and control groups. **b**. Power of GAMLSS-BEZI vs. LMAS for different effect sizes of differential relative abundances between case and control groups. GAMLSS-BEZI: Generalized Additive Models for Location, Scale and Shape (GAMLSS) with a zero inflated beta (BEZI) family; LMAS: linear model with arcsin squareroot transformation (implemented in the software MaAsLin); ROC curve: Receiver operating characteristic curve; AUC: area under the curve

Performance of GAMLSS-BEZI and LMAS was evaluated based on the receiver operating characteristic (ROC) curve for identifying species with differential abundance between case and control groups. The analysis for the ROC curves and area under the curve (AUC) was done using the R package ‘pROC’ version 1.10.0. Under these settings, GAMLSS-BEZI (AUC = 95.6, 95% CI = [94.2, 97.1%]) significantly outperformed LMAS (AUC = 92.9, 95% CI = [91.1, 94.7%]) (DeLong’s test *p*-value < 2.2e-16) (Fig. 1a).

We also performed simulations to evaluate power of GAMLSS-BEZI vs. LMAS for different effect sizes of differential relative abundances between case and control groups. Three settings for differential relative abundances (effect sizes) of one bacterial species were considered: 1) μ_1_ = 0.5 vs. μ_2_ = 0.4; 2) μ_1_ = 0.5 vs. μ_2_ = 0.3; and 3) μ_1_ = 0.5 vs. μ_2_ = 0.2. Other parameters were set the same for case and control groups (α_1_ = α_2_ = 0.5, ϕ_1_= ϕ_2_ = 5). A sample size of *n* = 100 for both case and control groups was used and the relative abundance of a bacterial species was simulated in each setting. The simulations were repeated 1000 times. Power of GAMLSS-BEZI or LMAS was calculated as the proportion of simulations with *p*-values of GAMLSS-BEZI or LMAS < 0.05 over the total number of 1000 simulations. Under these settings, power of GAMLSS-BEZI was better than power of LMAS (Fig. 1b).

### Performance of GAMLSS- BEZI: application to real microbiome data

#### Type I error

We evaluated the type I error of GAMLSS-BEZI and LMAS using published data from a cohort study of 50 healthy Bangladeshi infants, which included longitudinal gut microbiome data from 996 stool samples collected monthly from birth to 2 years of life [14]. We used data from a subset of samples collected around birth as a cross-sectional dataset (50 samples) and data from all samples as a longitudinal dataset (996 samples). For each dataset, we randomly split the samples into two groups (case vs. control) and compared relative abundances of all bacterial taxa at all taxonomic levels (272 taxa from phylum to genus levels in total) between these two random groups using GAMLSS-BEZI and LMAS. The procedure was repeated 1000 times. Type I error was calculated for three different alpha levels of 0.01, 0.05 and 0.1. For each taxon, the type I error of GAMLSS-BEZI or LMAS was defined as the proportion of random splits with *p*-values of GAMLSS-BEZI or LMAS less than the corresponding alpha level over 1000 random splits. We noted that type I errors were well controlled in both GAMLSS-BEZI and LMAS (Table 2).

**Table 2:** Type I error of GAMLSS-BEZI and LMAS on real microbiome data

Taxonomic level	GAMLSS-BEZI	LMAS	GAMLSS-BEZI	LMAS	GAMLSS-BEZI	LMAS
	Alpha level = 0.01 (median (IQR))		Alpha level = 0.05 (median (IQR))		Alpha level = 0.1 (median (IQR))	
Cross-sectional microbiome data						
Phylum (5 taxa)	0.010 (0.007, 0.017)	0.007 (0.003, 0.010)	0.043 (0.043, 0.050)	0.040 (0.033, 0.043)	0.100 (0.093, 0.113)	0.090 (0.073, 0.090)
Family (33 taxa)	0.000 (0.000, 0.003)	0.000 (0.000, 0.007)	0.007 (0.000, 0.043)	0.033 (0.007, 0.050)	0.070 (0.003, 0.103)	0.083 (0.053, 0.107)
Longitudinal microbiome data						
Phylum (5 taxa)	0.007 (0.002, 0.012)	0.010 (0.008, 0.013)	0.047 (0.030, 0.060)	0.067 (0.063, 0.080)	0.110 (0.075, 0.123)	0.117 (0.113, 0.132)
Family (33 taxa)	0.003 (0.000, 0.008)	0.010 (0.007, 0.013)	0.043 (0.036, 0.053)	0.050 (0.043, 0.064)	0.097 (0.082, 0.110)	0.107 (0.089, 0.117)

*GAMLSS-BEZI* Generalized Additive Models for Location, Scale and Shape (GAMLSS) with a zero inflated beta (BEZI) family, *LMAS* linear model with arcsin square root transformation (implemented in the software MaAsLin); *IQR* interquartile range. For longitudinal data, subject random intercepts were added to the models

#### Computation time

The running time of GAMLSS-BEZI for testing all bacterial taxa at all taxonomic levels from phylum to genus (272 taxa in total) on a standard laptop were 6.4 s for the cross-sectional dataset (50 samples) and 12.4 s for the longitudinal dataset (996 samples), respectively. This indicates that the GAMLSS-BEZI algorithm is computationally efficient.

#### Detecting differential abundance

We evaluated the performance of GAMLSS-BEZI vs. LMAS in detecting differential relative abundances using published data from a cohort study of 50 healthy Bangladeshi infants described above [14]. This study included longitudinal monthly data regarding the infants’ breastfeeding practices (exclusive, non-exclusive), duration of exclusive breastfeeding, infant age (months) at solid food introduction, and occurrence of diarrhea around the time of stool sample collection. We compared the performance of GAMLSS-BEZI vs. LMAS in detecting differential relative abundances between various grouping variables in three examples below.

*Example 1: Comparison of longitudinal monthly gut bacterial relative abundances at phylum level between non-exclusively breastfed (non-EBF)* vs. *exclusively breastfed (EBF) infants from birth to ≤ 6 months of age*

**Fig. 2:**
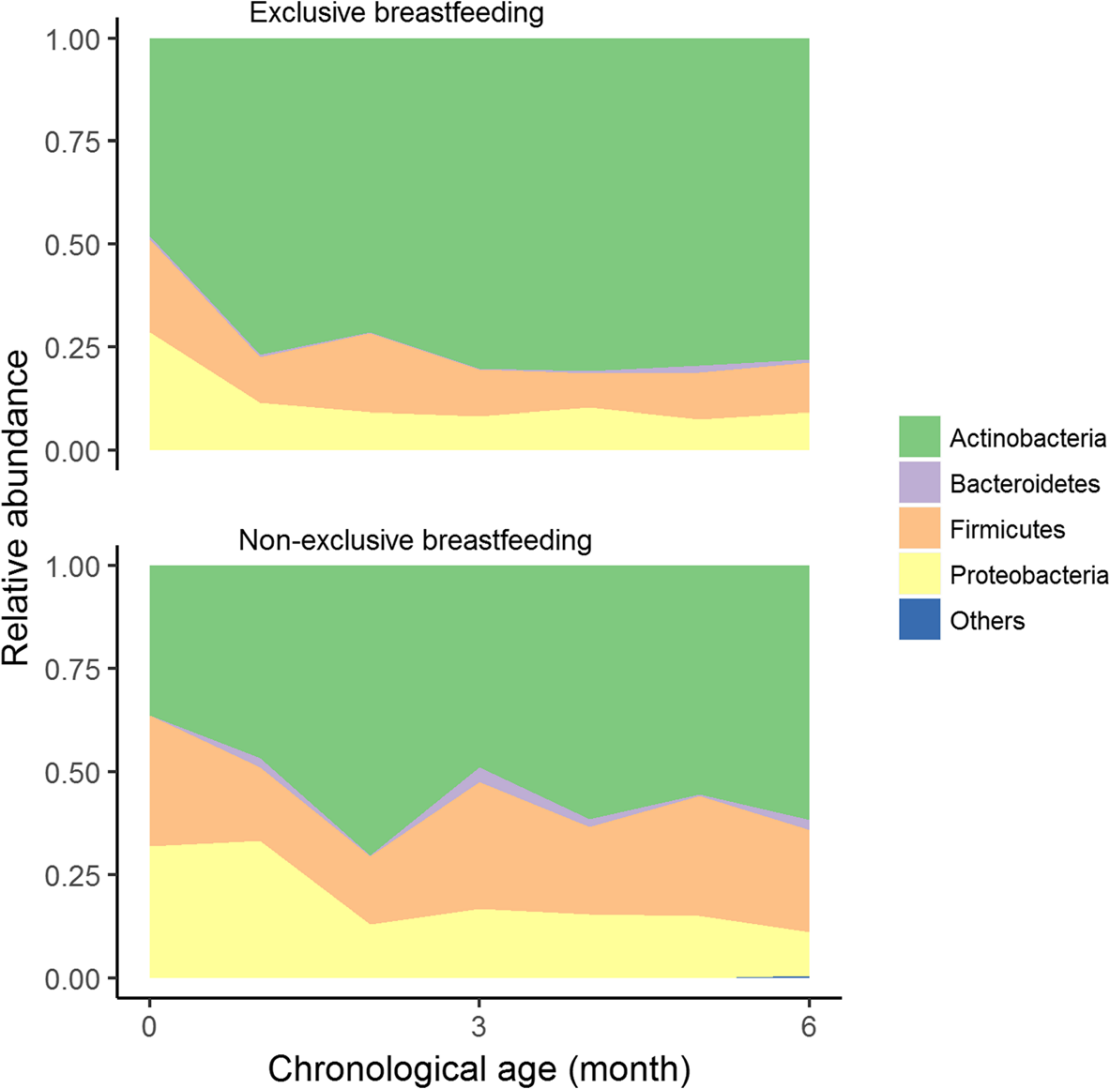
Relative abundances of bacterial phyla in non-exclusively breastfed vs. exclusively breastfed infants ≤6 months of age. Data from Bangladesh study

Figure 2 (produced using the function *taxa.mean.plot* of our ‘*metamicrobiomeR’* package; more details in Additional file 1) shows the longitudinal monthly average of relative abundance of bacterial phyla in non-EBF and EBF infants from birth to 6 months of age. A higher abundance of Proteobacteria, Firmicutes, and Bacteroidetes as well as a lower abundance of Actinobacteria are observed in non-EBF versus EBF infants. GAMLSS-BEZI is able to detect a significant difference in all four of these phyla whereas LMAS can only detect a significant difference in three phyla (Table 3).

**Table 3:** Results of GAMLSS-BEZI and LMAS: real microbiome data example 1

	GAMLSS-BEZI					LMAS				
Bacterial phyla	Estimate	95% Lower limit	95% Upper limit	*p*-value	FDR adjusted *p*-value	Estimate	95% Lower limit	95% Upper limit	*p*-value	FDR adjusted *p*-value
Actinobacteria	−0.37	− 0.65	− 0.10	**0.0083**	0.0166	−0.13	− 0.23	− 0.03	**0.0088**	0.0207
Bacteroidetes	0.26	0.00	0.53	**0.0499**	0.0499	0.03	0.00	0.05	**0.0292**	0.0390
Firmicutes	0.24	0.00	0.47	**0.0468**	0.0499	0.07	0.00	0.14	0.0668	0.0668
Proteobacteria	0.37	0.11	0.64	**0.0053**	0.0166	0.10	0.02	0.17	**0.0103**	0.0207

Data from Bangladesh study. Comparison of longitudinal monthly gut bacterial relative abundances at phylum level between non-exclusively breastfed (non-EBF) vs. exclusively breastfed (EBF) infants from birth to ≤6 months of age using GAMLSS-BEZI vs. LMAS. Significant *p*-values (< 0.05) are in bold

*GAMLSS-BEZI* Generalized Additive Models for Location, Scale and Shape (GAMLSS) with a zero inflated beta (BEZI) family, *LMAS* linear model with arcsin square root transformation (implemented in the software MaAsLin), *FDR* false discovery rate

*Example 2: Comparison of longitudinal monthly gut bacterial relative abundances at phylum level between infants from 6 months to 2 years of age introduced to solid food after 5 months vs. before 5 months*

**Fig. 3:**
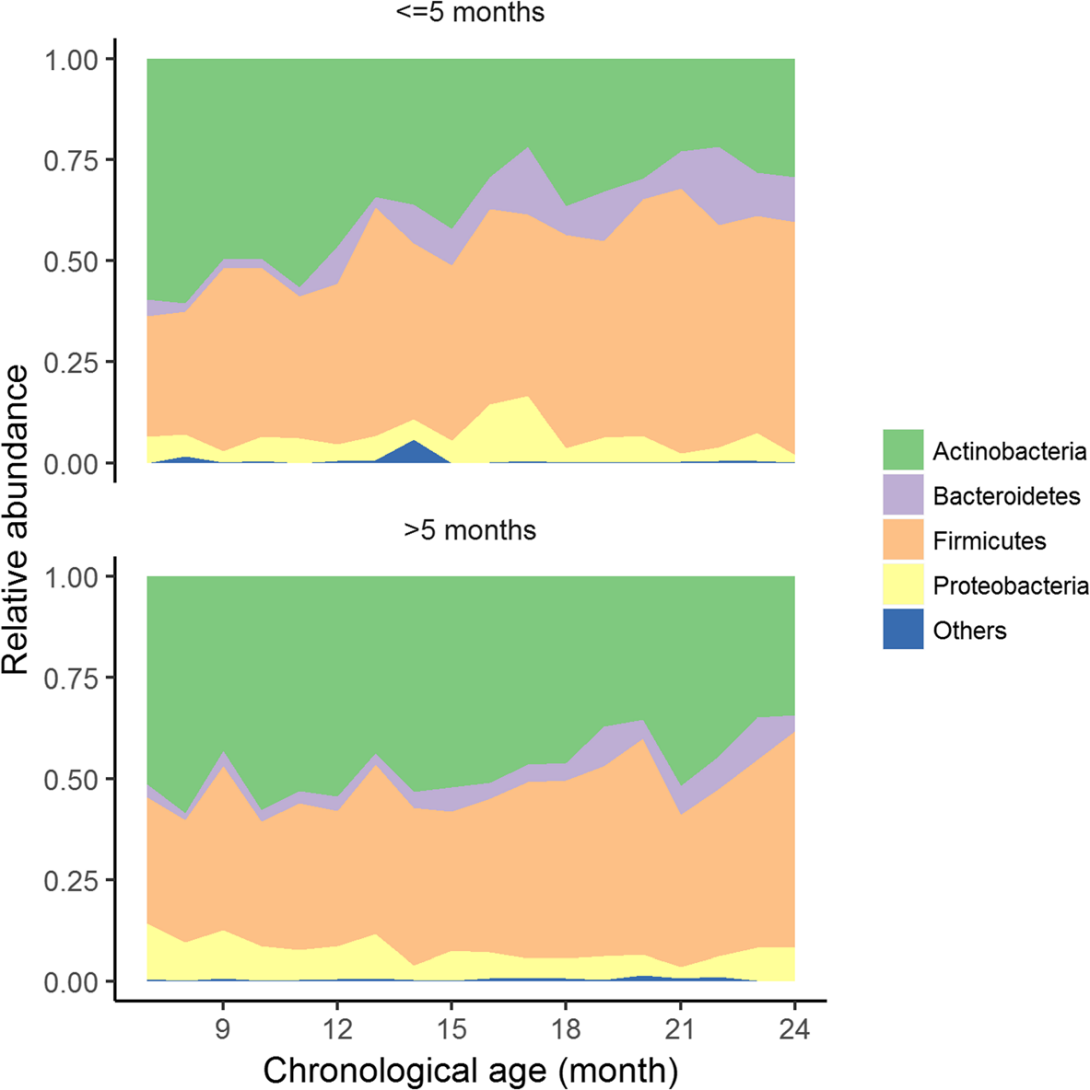
Relative abundances of bacterial phyla in infants from 6 months to 2 years of age with solid food introduction after 5 months vs. before 5 months. Data from Bangladesh study

Figure 3 shows the longitudinal monthly average of relative abundance of bacterial phyla in two groups of infants from 6 months to 2 years of age who were introduced to solid food after 5 months vs. those before 5 months of life. Lower relative abundances of Firmicutes, Bacteroidetes and higher relative abundance of Actinobacteria are observed in infants with solid food introduction after 5 months. GAMLSS-BEZI detects all three of these differences whereas LMEM can only detect a significant difference in one phylum (Table 4).

**Table 4:** Results of GAMLSS-BEZI and LMAS: real microbiome data example 2

	GAMLSS-BEZI					LMAS				
Bacterial phyla	Estimate	95% Lower limit	95% Upper limit	*p*-value	FDR adjusted *p*-value	Estimate	95% Lower limit	95% Upper limit	*p*-value	FDR adjusted *p*-value
Actinobacteria	0.19	0.04	0.34	**0.0119**	0.0208	0.05	−0.06	0.16	0.3451	0.3451
Bacteroidetes	−0.26	−0.42	− 0.10	**0.0018**	0.0070	−0.05	− 0.09	−0.01	**0.027**	0.1079
Firmicutes	−0.16	−0.30	− 0.03	**0.0156**	0.0208	−0.04	− 0.12	0.04	0.3168	0.3451
Proteobacteria	0.14	−0.02	0.30	0.0861	0.0861	0.02	−0.02	0.07	0.2916	0.3451

Data from Bangladesh study. Comparison of longitudinal monthly gut bacterial relative abundances at phylum level between infants from 6 months to 2 years of age with solid food introduction after 5 months vs. before 5 months of age using GAMLSS-BEZI vs. LMAS. Significant *p*-values (< 0.05) are in bold

*GAMLSS-BEZI* Generalized Additive Models for Location, Scale and Shape (GAMLSS) with a zero inflated beta (BEZI) family, *LMAS* linear model with arcsin square root transformation (implemented in the software MaAsLin), *FDR* false discovery rate

Example 1 and 2 demonstrate the increased sensitivity of GAMLSS-BEZI in detecting bacterial taxa with observed differential relative abundances as compared to LMAS.

*Example 3: Comparison of longitudinal monthly gut bacterial relative abundances at phylum level in infants from 6 months to 2 years of age with* vs. *without diarrhea stratified by duration of exclusive breastfeeding (EBF)*

**Fig. 4:**
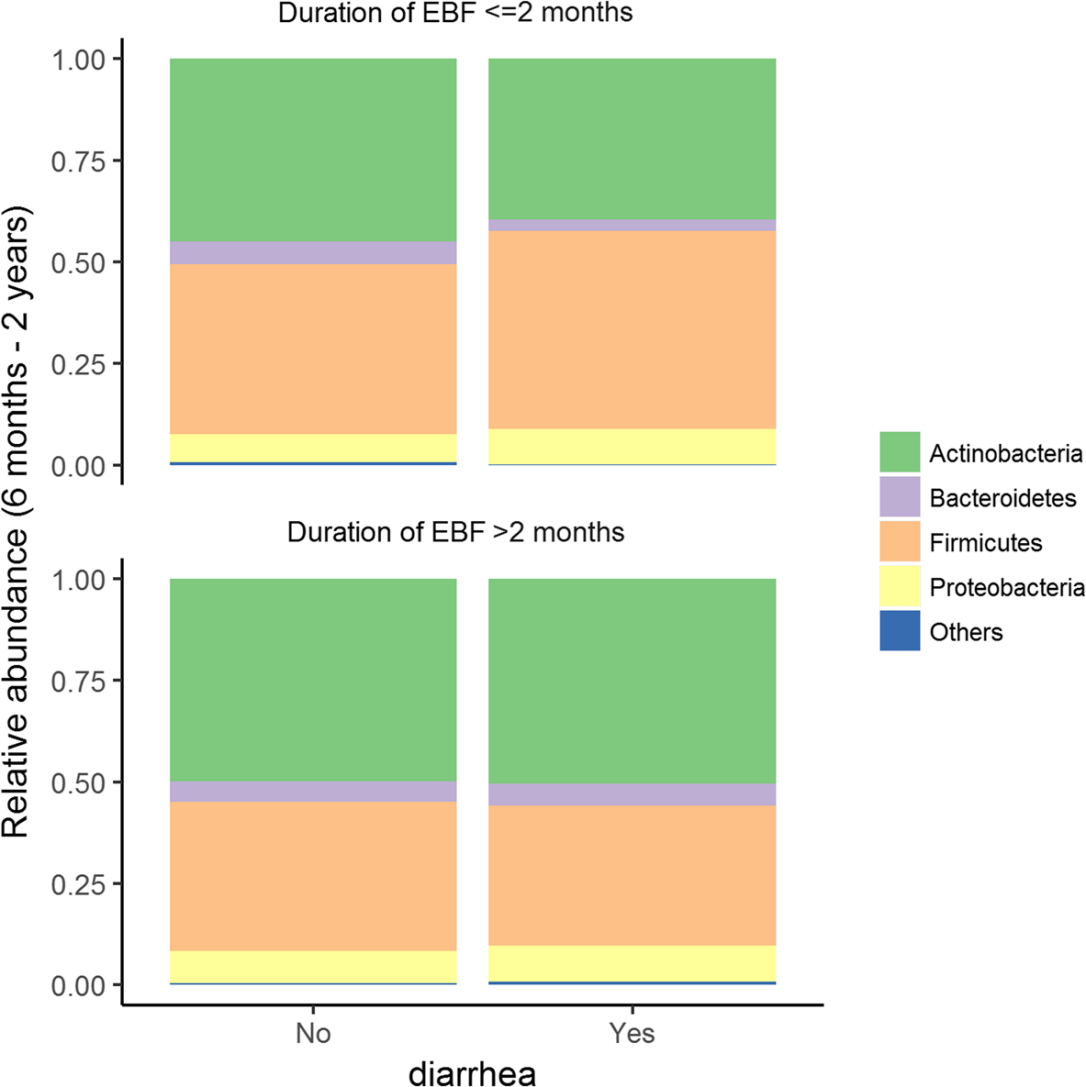
Relative abundance of bacterial phyla in infants from 6 months to 2 years of age with diarrhea vs. without diarrhea at the time of stool sample collection stratified by duration of exclusive breastfeeding (EBF). Data from Bangladesh study

Figure 4 shows the average of relative abundance of bacterial phyla in groups of infants from 6 months to 2 years of age with vs. without diarrhea around the time of stool sample collection stratified by duration of EBF. In infants who received less than two months of EBF, a higher abundance of Firmicutes and a lower abundance of Actinobacteria is observed in the groups of infants with diarrhea vs. those without diarrhea (Fig. 4, upper panel). GAMLSS-BEZI detects a significant difference in both Firmicutes and Actinobacteria. In contrast, in infants who received more than two months of EBF, no difference in relative abundance of any bacterial phylum is observed between those with diarrhea vs. those without diarrhea (Fig. 4, lower panel) and GAMLSS-BEZI does not report any significant difference (Table 5). This example demonstrates that GAMLSS-BEZI detects differential abundances when there is observed difference and does not report difference when there is no observed difference.

**Table 5:** Results of GAMLSS-BEZI and LMAS: real microbiome data example 3

	GAMLSS-BEZI					LMAS				
Bacterial phyla	Estimate	95% Lower limit	95% Upper limit	*p*-value	FDR adjusted *p*-value	Estimate	95% Lower limit	95% Upper limit	*p*-value	FDR adjusted *p*-value
In infants with duration of EBF ≤ 2 months (diarrhea vs. no diarrhea comparison)										
Actinobacteria	−0.73	−1.12	−0.34	**0.0003**	0.0011	−0.12	−0.23	0.0	**0.0424**	0.0848
Bacteroidetes	−0.29	−0.68	0.10	0.1524	0.2032	0.06	−0.12	0.01	0.0852	0.1136
Firmicutes	0.49	0.15	0.84	**0.0055**	0.0109	0.11	0.01	0.2	**0.0269**	0.0848
Proteobacteria	−0.17	−0.54	0.20	0.3729	0.3729	0.00	−0.07	0.08	0.9060	0.9060
In infants with duration of EBF > 2 months (diarrhea vs. no diarrhea comparison)										
Actinobacteria	0.02	−0.42	0.46	0.9243	0.9243	0.00	−0.10	0.10	0.9626	0.9989
Bacteroidetes	0.07	−0.41	0.56	0.7680	0.9243	0.01	−0.07	0.09	0.8101	0.9707
Firmicutes	−0.02	−0.40	0.36	0.9142	0.9243	−0.01	−0.13	0.12	0.8927	0.9707
Proteobacteria	0.12	−0.33	0.56	0.6043	0.9243	0.02	−0.06	0.11	0.5875	0.9191

Data from Bangladesh study. Comparison of longitudinal monthly gut bacterial relative abundances at phylum level in infants from 6 months to 2 years of age with diarrhea vs. no diarrhea at the time of stool sample collection stratified by duration of exclusive breastfeeding (EBF). Significant *p*-values (< 0.05) are in bold

*EBF* exclusive breastfeeding, *GAMLSS-BEZI* Generalized Additive Models for Location, Scale and Shape (GAMLSS) with a zero inflated beta (BEZI) family, *LMAS* linear model with arcsin squareroot transformation (implemented in the software MaAsLin); *FDR* false discovery rate

### Illustration of meta-analysis examples with real microbiome data from four studies

We used gut microbiome data from four published studies to demonstrate the application of random effects models for meta-analysis across microbiome studies. These four studies include: 1) a cohort of healthy infants in Bangladesh [14] (the data of this study was also used in the three examples demonstrating the performance of GAMLSS-BEZI above); 2) a cross-sectional study of Haiti infants negative for HIV who were exposed or unexposed to maternal HIV [11]; 3) a cohort of healthy infants in the USA (California and Florida [CA_FL]) [12]; and 4) a small cohort of healthy infants in the USA (North Carolina [NC]) [35]. More details about the four studies included in the meta-analysis are described in Table 6. We illustrate the example of meta-analysis comparing relative abundances of gut bacterial taxa and bacterial predicted functional pathways between male vs. female infants ≤6 months of age adjusting for feeding status and infant age at the time of stool sample collection across these four studies (total number of stool samples = 610 [female = 339, male = 271]).

**Table 6:** Summary of four published microbiome studies included in meta-analysis

Published study	Data origin (study population)	Study design/ Data used in meta-analysis	Sample size (for only infants ≤ 6 months of age)	Clinical variables used in meta-analysis	Target region of 16S rRNA genes /sequence platform	Starting files used and data processing done in this project
Subramanian et al. (2014). Persistent gut microbiota immaturity in malnourished Bangladeshi children [14]	Bangladesh^b^	Longitudinal gut microbiome data from stool samples collected monthly from birth to 6 months of age of 50 healthy Bangladeshi infants (25 singletons, 11 twin pairs, 1 set of triplets).^a^	Number of samples: 322 (female =180, male =142)	Gender, feeding status (EBF, non-EBF, non-BF), infant age at sample collection	V4 /Illumina MiSeq	Assembled 16S reads used for OTU picking (.fna file), mapping and meta-data files. - Open OTU picking with UCLUST with 97% similarity using the Greengenes database (version 13.8)
Bender et al. (2016). Maternal HIV infection influences the microbiome of HIV- uninfected infants [11]	Haiti	One time gut microbiome data from stool samples of 48 HIV negative infants with age varied from 0 to 6 months whose mothers were HIV negative (*n* = 25) or HIV positive (*n* = 23).	Number of samples: 48 (female =25, male =21)	Gender, feeding status (EBF, non-EBF), infant age at sample collection	V4 /Illumina MiSeq	
Pannaraj et al. (2017). Association Between Breast Milk Bacterial Communities and Establishment and Development of the Infant Gut Microbiome [12]	USA (California and Florida)	Longitudinal gut microbiome data from stool samples of 113 healthy full-term infants collected at 0 to 7 days, 8 to 30 days, 31 to 90 days, 91 to 180 days.	Number of samples: 221 (female = 120, male = 101)	Gender, feeding status (EBF, non-EBF, non-BF), infant age at sample collection	V4 /Illumina MiSeq	
Thompson et al. (2015). Milk- and solid-feeding practices and daycare attendance are associated with differences in bacterial diversity, predominant communities, and metabolic and immune function of the infant gut microbiome [35]	USA (North Carolina)	Longitudinal gut microbiome data from stool samples of 6 healthy full term infants with age varied from 0 to 6 months.	Number of samples: 21 (female = 14, male = 7)	Gender, feeding status (EBF, non-EBF, non-BF), infant age at sample collection	V1–2 /Roche GS FLX Titanium	

^a^This healthy cohort was used as reference in the comparison with malnourished cohorts in the original published paper. ^b^The healthy cohort of this Bangladesh study also contain 674 stool samples > 6 months of age. The data of this healthy cohort were also used in the analyses comparing the performance of GAMLSS-BEZI (Generalized Additive Models for Location, Scale and Shape (GAMLSS) with a zero inflated beta (BEZI) family) vs. LMAS (linear model with arcsin squareroot transformation) in example 1, 2, 3 above. Data from this study was downloaded from the authors’ website: https://gordonlab.wustl.edu/Subramanian_6_14/Nature_2014_Processed_16S_rRNA_datasets.html. Data from three other studies were obtained directly from the investigators. EBF: exclusive breastfeeding; non-EBF: non-exclusive breastfeeding; non-BF: non-breastfeeding

#### Relative abundances of gut bacterial taxa

**Fig. 5:**
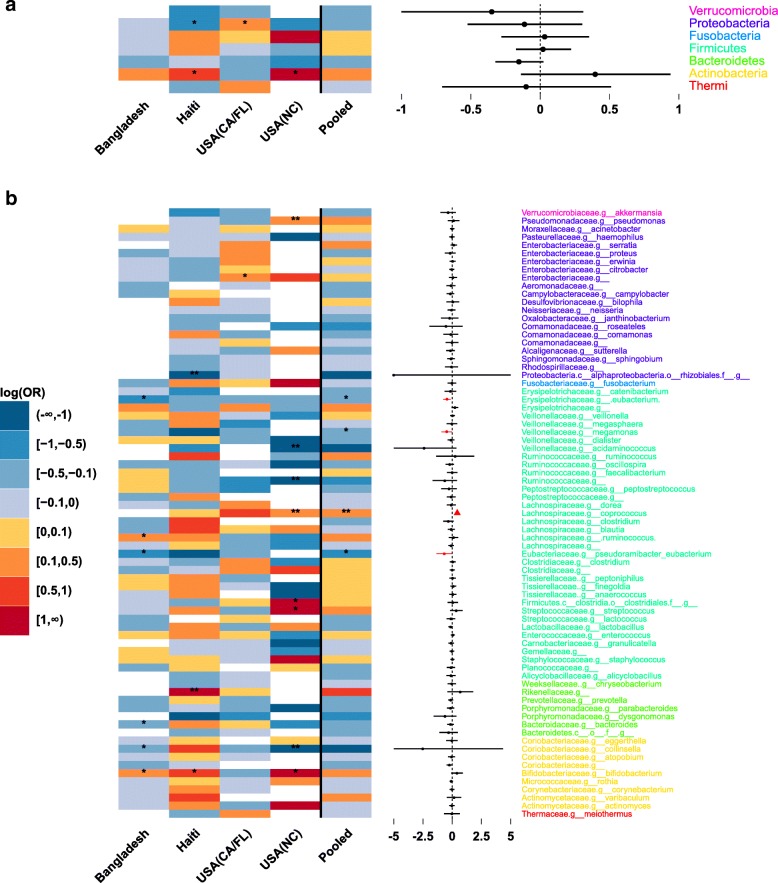
Meta-analysis for the difference in relative abundances of gut bacterial taxa between male vs. female infants ≤6 months of age. **a**: Phylum level: heatmap of log (odds ratio) (log (OR)) of relative abundances of all gut bacterial phyla between male vs. female infants for each study and forest plot of pooled estimates across all studies with 95% confidence intervals (95% CI). **b**: Genus level: heatmap of log (OR) of relative abundances of all gut bacterial genera between male vs. female infants for each study and forest plot of pooled estimates across all studies with 95% CI. All log (OR) estimates of each bacterial taxa from each study were from Generalized Additive Models for Location Scale and Shape (GAMLSS) with beta zero inflated family (BEZI) and were adjusted for feeding status and age of infants at sample collection. Pooled log (OR) estimates and 95% CI (forest plot) were from random effect meta-analysis models with inverse variance weighting and DerSimonian–Laird estimator for between-study variance based on the adjusted log (OR) estimates and corresponding standard errors of all included studies. Bacterial taxa with *p*-values for differential relative abundances < 0.05 are denoted with * and those with p-values < 0.0001 are denoted with **. Pooled log (OR) estimates with pooled p-values< 0.05 are in red and those with false discovery rate (FDR) adjusted pooled p-values < 0.1 are shown as triangles. Missing (unavailable) values are in white. USA: United States of America; CA: California; FL: Florida; NC: North Carolina

Meta-analysis results are visually displayed using the functions *metatab.show* and *meta.niceplot* of our *‘metamicrobiomeR’* package (Additional file 1). The adjusted estimates (log (odds ratio) of one gender group for changes in relative abundance) from GAMLSS-BEZI for each bacterial taxon of each of the four studies and the pooled adjusted estimates across studies (meta-analysis) are displayed as a heatmap (Fig. 5 left panel). Different significant levels of *p*-values are denoted for each taxon of each study. The adjacent forest plot displays the pooled adjusted estimates and their 95% CI with different colors and shapes to reflect the magnitude of pooled p-values (Fig. 5 right panel).

The running time for meta-analysis using both random effects and fixed effects models across four studies for all bacterial taxa (328 taxa available in at least 2 studies) from phylum to genus levels was 3.7 s on a standard laptop. This indicates that the meta-analysis algorithm is computationally efficient.

Across the four studies, there is a large heterogeneity in the difference (log (odds ratio)) of gut bacterial taxa relative abundances between male vs. female infants ≤6 months of age after adjusting for feeding status and age of infants at sample collection (Fig. 5, Additional file 1). For example, at the phylum level, relative abundance of Actinobacteria is significantly higher in male vs. female infants in two studies with small sample sizes (Haiti and North Carolina) while two other studies with larger sample size (Bangladesh and US (CA_FL) shows non-significant results in opposite directions. In addition, differential relative abundance of Proteobacteria is significant in two studies but in opposite directions (higher in male infants in the USA (CA_FL) study while lower in male infants in the Haiti study as compared to female infants). Moreover, at the genus level, each study shows significant differential relative abundances of different bacterial genera between male vs. female infants and the effects of many genera are in opposite directions between studies. Since the results are heterogeneous or opposite between studies and thus difficult to interpret, meta-analysis across studies is necessary to evaluate the overall consistent effects.

On the other hand, there are also some consistent effects across studies. For example, phylum Bacteroidetes is consistently decreased in male vs. female infants across four studies. However, the decrease is not significant in any study (Fig. 5a). Therefore, meta-analysis across studies is also important to evaluate if there is an overall significant effect.

Meta-analysis of the four studies shows no significant differential relative abundance of any bacterial phylum between male vs. female infants (Fig. 5a). At the genus level, meta-analyses show four genera with significant consistent differential relative abundances (pooled *p*-value < 0.05) between male vs. female infants. After adjusting for multiple testing, only genus *Coprococcus* remains significantly higher in male vs. female infants (FDR adjusted pooled p-value< 0.0001) (Fig. 5b).

#### Relative abundances of bacterial predicted functional (KEGG) pathways

**Fig. 6:**
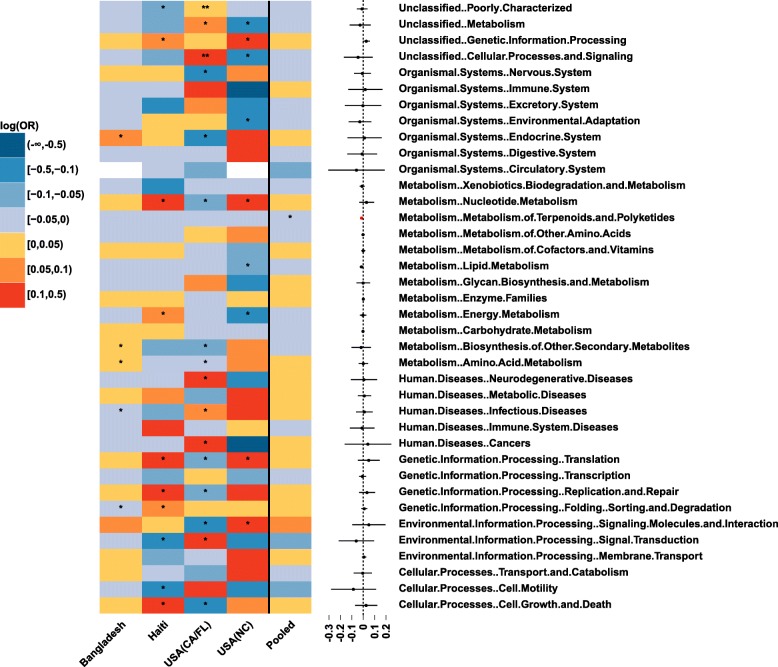
Meta-analysis for the difference in relative abundances of gut microbial KEGG pathways between male vs. female infants ≤6 months of age. Heatmap of log (odds ratio) (log (OR)) of relative abundances of gut microbial KEGG pathways at level 2 between male vs. female infants for each study and forest plot of pooled estimates of all studies with 95% confidence intervals (95% CI). All log (OR) estimates of each pathway from each study were from Generalized Additive Models for Location Scale and Shape (GAMLSS) with beta zero inflated family (BEZI) and were adjusted for feeding status and age of infants at sample collection. Pooled log (OR) estimates and 95%CI (forest plot) were from random effect meta-analysis models with inverse variance weighting and DerSimonian–Laird estimator for between-study variance based on the adjusted log (OR) estimates and corresponding standard errors of all included studies. Pathways with *p*-values for differential relative abundances < 0.05 are denoted with * and those with *p*-values < 0.0001 are denoted with **. Pooled log (OR) estimates with pooled p-values< 0.05 are in red and those with false discovery rate (FDR) adjusted pooled p-values < 0.1 are shown as triangles. KEGG: Kyoto Encyclopedia of Genes and Genomes; USA: United States of America; CA: California; FL: Florida; NC: North Carolina

Across the four studies, there is also a large heterogeneity in the difference (log (odds ratio)) of relative abundances of gut bacterial predicted functional KEGG pathways between male vs. female infants ≤6 months of age after adjusting for feeding status and age of infants at sample collection (Fig. 6). For example, at level 2 of KEGG pathway, the USA (CA_FL) study (with relatively large sample size) shows many pathways with significant differential relative abundances between male vs. female infants. The other three studies varyingly show significantly differential relative abundances in some of these pathways. However, the effects of almost all of these pathways in the USA (CA_FL) study are in opposite directions with the effects of these pathways in any of the other three studies. Therefore, it is difficult to interpret the results regarding male vs. female pathway differential relative abundances. As such, meta-analysis across studies is important to examine the overall consistent effects. Meta-analysis of four included studies shows only one KEGG pathway at level 2 with significant consistent differential relative abundance between male vs. female infants (pooled *p*-value < 0.05). However, after adjusting for multiple testing, no KEGG pathway (at both level 2 and level 3) remains significantly different between genders (Fig. 6, Additional file 1).

Difference in gut microbial composition between genders has been reported in adults [36, 37] and in some neonatal studies albeit with small sample sizes [38, 39]. However, the reported findings have largely varied between these studies. Our analyses also showed heterogeneous results among the four studies included. This highlights the importance of meta-analyses to evaluate overall consistent results across studies. Our meta-analyses of four studies showed virtually no difference in gut bacterial community and predicted functional pathways between male vs. female infants’ ≤6 months of age after adjusting for feeding status and infant age at time of sample collection as well as after adjusting for multiple testing. There was one exception: relative abundance of *Coprococcus* was significantly higher in male vs. female infants. *Coprococcus* has been implicated in many conditions including hypertension and autism [40, 41], and the detected difference in our study may provide some insights into the known sex differences in health outcomes.

In addition, random effects meta-analysis models can also be generally applied to other microbiome measures such as microbial alpha diversity and microbiome age. To make the estimates for these positive continuous microbiome measures comparable across studies, these measures should be standardized to have a mean of 0 and standard deviation of 1 before between-group-comparison within each study. Random effects meta-analysis models can then be applied to pool the “comparable” estimates and their standard errors across studies. Meta-analysis results of these measures can be displayed as standard meta-analysis forest plots (Additional file 1).

## Conclusion

Our *metamicrobiomeR* package implemented GAMLSS-BEZI for analysis of microbiome relative abundance data and random effects meta-analysis models for meta-analysis across microbiome studies. The advantages of GAMLSS-BEZI are: 1) it directly address the distribution of microbiome relative abundance data which resemble a zero-inflated beta distribution; 2) it has better power to detect differential relative abundances between groups than the commonly used approach LMAS; 3) the estimates from GAMLSS-BEZI are log (odds ratio) of relative abundances of bacterial taxa between comparison groups and thus are directly analogous across studies. Random effects meta-analysis models can be directly applied to pool the adjusted estimates and their standard errors across studies. This approach allows examination of study-specific effects, heterogeneity between studies, and the overall pooled effects across microbiome studies. The examples and workflow using our “*metamicrobiomeR”* package are reproducible and applicable for the analysis and meta-analysis of other microbiome studies. The R package *‘metamicrobiomeR’* we developed will help researchers to readily conduct microbiome meta-analysis appropriately.

## Availability and requirements

Project name: metamicrobiomeR.

Project home page: https://github.com/nhanhocu/metamicrobiomeR

Operating system(s): Platform independent.

Programming language: R.

Other requirements: R 3.4.2 or higher.

License: GNU GPL v. 2.

Any restrictions to use by non-academics: none.

## Additional file

Additional file 1: A summary of implemented functions and tutorial for the ‘metamicrobiomeR’ package. (HTML 2364 kb)

## Abbreviations

BEZI: Zero inflated beta
CA: California
CI: Confidence interval
CLR: Centered Log Ratio
EBF: Exclusive breastfeeding (or exclusively breastfed)
FDR: False Discovery Rate
FL: Florida
GAMLSS: Generalized Additive Models for Location, Scale and Shape
GAMLSS-BEZI: Generalized Additive Models for Location, Scale and Shape with a zero inflated beta family
GMPR: Geometric Mean of Pairwise Ratios
KEGG: Kyoto Encyclopedia of Genes and Genomes
LM: Linear/linear mixed effect models
LMAS: Linear/linear mixed effect models with arcsin squareroot transformation
NC: North Carolina
Non- EBF: Non exclusive breastfeeding (or non exclusively breastfed)
RAIDA: Ratio Approach for Identifying Differential Abundance
USA: United States of America
ZIL: Zero-inflated lognormal
ZINB: Zero-inflated negative model

## References

[1] Kim D, Hofstaedter CE, Zhao C, Mattei L, Tanes C, Clarke E (2017). Optimizing methods and dodging pitfalls in microbiome research. Microbiome..

[2] Sinha R, Abu-Ali G, Vogtmann E, Fodor AA, Ren B, Amir A (2017). Assessment of variation in microbial community amplicon sequencing by the microbiome quality control (MBQC) project consortium. Nat Biotechnol.

[3] Adams RI, Bateman AC, Bik HM, Meadow JF (2015). Microbiota of the indoor environment: a meta-analysis. Microbiome..

[4] Bhute S, Pande P, Shetty SA, Shelar R, Mane S, Kumbhare SV (2016). Molecular characterization and meta-analysis of gut microbial communities illustrate enrichment of Prevotella and Megasphaera in Indian subjects. Front Microbiol.

[5] Holman DB, Brunelle BW, Trachsel J, Allen HK (2017). Meta-analysis To Define a Core Microbiota in the Swine Gut. mSystems.

[6] Mancabelli L, Milani C, Lugli GA, Turroni F, Ferrario C, van Sinderen D (2017). Meta-analysis of the human gut microbiome from urbanized and pre-agricultural populations. Environ Microbiol.

[7] Lozupone CA, Stombaugh J, Gonzalez A, Ackermann G, Wendel D, Vázquez-Baeza Y (2013). Meta-analyses of studies of the human microbiota. Genome Res.

[8] Duvallet C, Gibbons SM, Gurry T, Irizarry RA, Alm EJ (2017). Meta-analysis of gut microbiome studies identifies disease-specific and shared responses. Nat Commun.

[9] Sze MA, Schloss PD (2016). Looking for a signal in the noise: revisiting obesity and the microbiome. MBio..

[10] Dhariwal A, Chong J, Habib S, King IL, Agellon LB, Xia J (2017). MicrobiomeAnalyst: a web-based tool for comprehensive statistical, visual and meta-analysis of microbiome data. Nucleic Acids Res.

[11] Bender JM, Li F, Martelly S, Byrt E, Rouzier V, Leo M (2016). Maternal HIV infection influences the microbiome of HIV-uninfected infants. Sci Transl Med.

[12] Pannaraj PS, Li F, Cerini C, Bender JM, Yang S, Rollie A (2017). Association between breast Milk bacterial communities and establishment and development of the infant gut microbiome. JAMA Pediatr.

[13] Caporaso JG, Kuczynski J, Stombaugh J, Bittinger K, Bushman FD, Costello EK (2010). QIIME allows analysis of high-throughput community sequencing data. Nat Methods.

[14] Subramanian S, Huq S, Yatsunenko T, Haque R, Mahfuz M, Alam MA (2014). Persistent gut microbiota immaturity in malnourished Bangladeshi children. Nature..

[15] Stewart CJ, Embleton ND, Clements E, Luna PN, Smith DP, Fofanova TY (2017). Cesarean or vaginal birth does not impact the longitudinal development of the gut microbiome in a cohort of exclusively preterm infants. Front Microbiol.

[16] Morgan XC, Tickle TL, Sokol H, Gevers D, Devaney KL, Ward DV (2012). Dysfunction of the intestinal microbiome in inflammatory bowel disease and treatment. Genome Biol.

[17] Sordillo JE, Zhou Y, McGeachie MJ, Ziniti J, Lange N, Laranjo N (2017). Factors influencing the infant gut microbiome at age 3–6 months: Findings from the ethnically diverse Vitamin D Antenatal Asthma Reduction Trial (VDAART). J Allergy Clin Immunol.

[18] Bajer L, Kverka M, Kostovcik M, Macinga P, Dvorak J, Stehlikova Z (2017). Distinct gut microbiota profiles in patients with primary sclerosing cholangitis and ulcerative colitis. World J Gastroenterol.

[19] Hall AB, Yassour M, Sauk J, Garner A, Jiang X, Arthur T (2017). A novel Ruminococcus gnavus clade enriched in inflammatory bowel disease patients. Genome Med.

[20] Larivière-Gauthier G, Thibodeau A, Letellier A, Yergeau É, Fravalo P (2017). Reduction of Salmonella shedding by sows during gestation in relation to its fecal microbiome. Front Microbiol.

[21] Paulson JN, Stine OC, Bravo HC, Pop M (2013). Differential abundance analysis for microbial marker-gene surveys. Nat Methods.

[22] Sohn MB, Du R, An L (2015). A robust approach for identifying differentially abundant features in metagenomic samples. Bioinformatics..

[23] Xu L, Paterson AD, Turpin W, Xu W (2015). Assessment and selection of competing models for zero-inflated microbiome data. PLoS One.

[24] Xia Y, Sun J (2017). Hypothesis testing and statistical analysis of microbiome. Genes Dis.

[25] Chen J, King E, Deek R, Wei Z, Yu Y, Grill D (2018). An omnibus test for differential distribution analysis of microbiome sequencing data. Bioinformatics..

[26] Rigby RA, Stasinopoulos DM (2005). Generalized additive models for location, scale and shape. J R Stat Soc.

[27] Chen EZ, Li H (2016). A two-part mixed-effects model for analyzing longitudinal microbiome compositional data. Bioinformatics..

[28] Langille MGI, Zaneveld J, Caporaso JG, McDonald D, Knights D, Reyes JA (2013). Predictive functional profiling of microbial communities using 16S rRNA marker gene sequences. Nat Biotechnol.

[29] McMurdie PJ, Holmes S (2014). Waste not, want not: why rarefying microbiome data is inadmissible. PLoS Comput Biol.

[30] Min Y, Agresti A (2005). Random effect models for repeated measures of zero-inflated count data. Stat Modelling.

[31] Aitchison J, Barceló-Vidal C, Martín-Fernández JA, Pawlowsky-Glahn V (2000). Logratio Analysis and Compositional Distance. Math Geol.

[32] Palarea-Albaladejo J, Martin-Fernandez JA (2015). zCompositions -- R package for multivariate imputation of left-censored data under a compositional approach. Chemom Intell Lab Syst.

[33] Chen L, Reeve J, Zhang L, Huang S, Wang X, Chen J (2018). GMPR: a robust normalization method for zero-inflated count data with application to microbiome sequencing data. PeerJ..

[34] Ospina R, Ferrari SLP (2012). A general class of zero-or-one inflated beta regression models. Comput Stat Data Anal.

[35] Thompson AL, Monteagudo-Mera A, Cadenas MB, Lampl ML, Azcarate-Peril MA (2015). Milk- and solid-feeding practices and daycare attendance are associated with differences in bacterial diversity, predominant communities, and metabolic and immune function of the infant gut microbiome. Front Cell Infect Microbiol.

[36] Haro C, Rangel-Zúñiga OA, Alcalá-Díaz JF, Gómez-Delgado F, Pérez-Martínez P, Delgado-Lista J (2016). Intestinal microbiota is influenced by gender and body mass index. PLoS One.

[37] Singh P, Manning SD (2016). Impact of age and sex on the composition and abundance of the intestinal microbiota in individuals with and without enteric infections. Ann Epidemiol.

[38] Martin R, Makino H, Cetinyurek Yavuz A, Ben-Amor K, Roelofs M, Ishikawa E (2016). Early-life events, including mode of delivery and type of feeding, siblings and gender, shape the developing gut microbiota. PLoS One.

[39] Cong X, Xu W, Janton S, Henderson WA, Matson A, McGrath JM (2016). Gut microbiome developmental patterns in early life of preterm infants: impacts of feeding and gender. PLoS One.

[40] Krajmalnik-Brown R, Lozupone C, Kang D-W, Adams JB (2015). Gut bacteria in children with autism spectrum disorders: challenges and promise of studying how a complex community influences a complex disease. Microb Ecol Health Dis.

[41] Li J, Zhao F, Wang Y, Chen J, Tao J, Tian G (2017). Gut microbiota dysbiosis contributes to the development of hypertension. Microbiome..

