# Supplementary material for: metamicrobiomeR: an R package for analysis of microbiome relative abundance data using zero-inflated beta GAMLSS and meta-analysis across studies using random effects models
Source: BMC Bioinformatics. 2019 Apr 16;20:188. doi: 10.1186/s12859-019-2744-2 (PMC6469060; doi:10.1186/s12859-019-2744-2)
Supplement: Supplementary file 1 — A summary of implemented functions and tutorial for the ‘metamicrobiomeR’ package. (HTML 2364 kb) [file 12859_2019_2744_MOESM1_ESM.html]

metamicrobiomeR: an R package for analysis of microbiome relative abundance data using zero inflated beta GAMLSS and meta-analysis across studies using random effects models


# metamicrobiomeR: an R package for analysis of microbiome relative abundance data using zero inflated beta GAMLSS and meta-analysis across studies using random effects models

#### *Nhan Thi Ho*

Columbia University Medical Center  

#### *March 04, 2019*

Abstract

This document illustrates the use of *metamicrobiomeR* package which implemented Generalized Additive Models for Location, Scale and Shape (GAMLSS) with zero inflated beta (BEZI) family for the analysis of microbiome relative abundance data and random effects meta-analysis models for pooling estimates across microbiome studies. Alongside, this document introduces comprehensive examples and workflow for the analyses of each microbiome study and meta-analysis pooling estimates across microbiome studies. **Keywords**: GAMLSS, zero inflated beta, meta-analysis, random effect, pooling estimates, microbiome, relative abundance.

# Introduction

In this document, we illustrate the use our R package *metamicrobiomeR* including:

1. The application of Generalized Additive Model for Location, Scale and Shape (GAMLSS)1 with beta zero inflated (BEZI) family for the analysis of microbiome relative abundance data. The GAMLSS with BEZI family allows examination of microbiome relative abundance data, which ranges from zero to one and is generally zero-inflated. This model also allows adjusting for covariates and can be used for longitudinal or non-longitudinal study design. In addition, the estimates from GAMLSS-BEZI are log(odds ratio) of relative abundances between groups and thus are comparable across studies and thus facilitate straightforward meta-analysis across studies in later stage. We showed some examples to illustrate the performance of the GAMLSS (in comparison with linear/linear mixed effect models (LM) and LM with arcsin squareroot transformation (implemented in MaAsLin software)) using gut microbiome data from the Bangladesh study of Subramanian et al.2 Their data was downloaded from the authors’ website. As additional options to address compositional effects, Geometric Mean of Pairwise Ratios (GMPR) normalization and centered log ratio (CLR) transformation of bacterial taxa composition with different zero-replacement procedures were also implemented.
2. The application of random effect meta-analysis models for pooling estimates across microbiome studies. This approach allows examination of study-specific effects, heterogeneity across studies and overall effects across studies. We introduced a comprehensive workflow for the analyses of each microbiome study and meta-analysis pooling estimates across microbiome studies. We showed examples for comparison of infant gut microbiome between genders adjusting for breastfeeding status and infant age at stool sample collection in infants <= 6 months. The gut microbiome data used in our examples were from four studies in Bangladesh, Haiti,3 USA(CA\_FL),4 and USA(UNC).5
3. In addition, we implemented the procedures for predicting microbiome age based on relative abundances of bacterial genera using Random Forest model. This was adapted from the original approach proposed by Subramanian et al. We also illustrate the use of linear mixed model (for longitudinal data) or linear model (for non-longitudinal data) for comparison of multiple alpha diversity indexes between groups adjusting for covariates.

# Implementation

The *metamicrobiomeR* package includes the functions below.

| Functions | Description |
| --- | --- |
| *taxa.filter* | Filter relative abundances of bacterial taxa or pathways using prevalence and abundance thresholds |
| *taxa.meansdn* | Summarize mean, standard deviation of abundances and number of subjects by groups for all bacterial taxa or pathways |
| *taxa.mean.plot* | Plot mean abundance by groups (from *taxa.meansdn* output) |
| *taxa.compare* | Compare relative abundances of bacterial taxa at all levels using GAMLSS or linear/linear mixed effect models (LM) or linear/linear mixed effect models with arcsin squareroot transformation (LMAS) |
| *pathway.compare* | Compare relative abundances of bacterial functional pathways at all levels using GAMLSS or LM or LMAS. Compare of log(absolute abundances) of bacterial functional pathways at all levels using LM |
| *taxcomtab.show* | Display the results of relative abundance comparison (from *taxa.compare* or *pathway.compare* outputs) |
| *meta.taxa* | Perform meta-analysis of relative abundance estimates of bacterial taxa or pathways (either from GAMLSS or LM or LMAS) across studies (from combined taxa.compare/pathway.compare outputs of all included studies) using random effect and fixed effect meta-analysis models |
| *metatab.show* | Display meta-analysis results of bacterial taxa or pathway relative abundances (from *meta.taxa* output) |
| *meta.niceplot* | Produce nice combined heatmap and forest plot for meta-analysis results of bacterial taxa and pathway relative abundances (from *metatab.show* output) |
| *read.multi* | Read multiple files in a path to R |
| *alpha.compare* | Calculate average alpha diversity indexes for a specific rarefaction depth, standardize and compare alpha diversity indexes between groups |
| *microbiomeage* | Predict microbiome age using Random Forest model based on relative abundances of bacterial genera shared with the Bangladesh study |

#### Install ‘metamicrobiomeR’ and other required packages

```
rm(list=ls()) # clear all
library(devtools)
#install and load package metamicrobiomeR
install_github("nhanhocu/metamicrobiomeR")
library(metamicrobiomeR) 
#Load other needed packages 
library(knitr)
library(plyr)
library(dplyr)
library(gdata)
library(gridExtra)
library(ggplot2)
library(lme4) 
library(lmerTest)
library(mgcv) 
library(meta)
```

# Results

## Performance of GAMLSS

### GAMLSS is more sensitive than LM or LMAS when there are observed difference

#### Example 1: Comparison between breastfeeding statuses in infants < 6 months of age

##### Plot of mean relative abundance by breastfeeding statuses and age at phylum level

```
data(taxtab.rm7)
taxlist.rm<-taxa.filter(taxtab=taxtab.rm[[5]],percent.filter = 0.05, relabund.filter = 0.00005)
taxa.meansdn.rm<-taxa.meansdn(taxtab=taxtab.rm[[5]],sumvar="bf",groupvar="age.sample")
taxa.meansdn.rm<-taxa.meansdn.rm[taxa.meansdn.rm$bf!="No_BF" &taxa.meansdn.rm$age.sample<=6,]
taxa.meansdn.rm$bf<-drop.levels(taxa.meansdn.rm$bf,reorder=FALSE)
#phylum
p.bf.l2<-taxa.mean.plot(tabmean=taxa.meansdn.rm,tax.lev="l2", comvar="bf", groupvar="age.sample",mean.filter=0.005, show.taxname="short")
p.bf.l2$p
```

##### Comparison between breastfeeding statuses adjusting for age of infants at sample collection using GAMLSS

```
# Comparison of bacterial taxa relative abundance using LMEM or GAMLSS (take some time to run). 
# Note: running time is not long in regular laptop for both analysis (~10s) and meta-analysis (~5s).  
# However, to save time making the tutorial, some saved data/results are loaded for downstream analysis/display.  
#taxacom6.zi.rmg<-taxa.compare(taxtab=taxtab6.rm[[5]],propmed.rel="gamlss",comvar="bf",adjustvar="age.sample",longitudinal="yes",p.adjust.method="fdr")
#load saved results 
data(taxacom6.rmg)
#phylum
kable(taxcomtab.show(taxcomtab=taxacom6.zi.rmg,tax.select=p.bf.l2$taxuse.rm, showvar="bfNon_exclusiveBF", tax.lev="l2",readjust.p=TRUE,p.adjust.method="fdr",p.cutoff = 1))
```

|  | id | Estimate.bfNon\_exclusiveBF | ll | ul | Pr(>|t|).bfNon\_exclusiveBF | pval.adjust.bfNon\_exclusiveBF |
| --- | --- | --- | --- | --- | --- | --- |
| 5 | k\_\_bacteria.p\_\_proteobacteria | 0.37 | 0.11 | 0.64 | 0.0053 | 0.0166 |
| 1 | k\_\_bacteria.p\_\_actinobacteria | -0.37 | -0.65 | -0.10 | 0.0083 | 0.0166 |
| 3 | k\_\_bacteria.p\_\_firmicutes | 0.24 | 0.00 | 0.47 | 0.0468 | 0.0499 |
| 2 | k\_\_bacteria.p\_\_bacteroidetes | 0.26 | 0.00 | 0.53 | 0.0499 | 0.0499 |

##### Comparison between breastfeeding statuses adjusting for age of infants at sample collection using LM (without transformation)

```
#taxacom6.rmg<-taxa.compare(taxtab=taxtab6.rm[[5]],propmed.rel="lm",comvar="bf",adjustvar="age.sample",longitudinal="yes",p.adjust.method="fdr")
#phylum
kable(taxcomtab.show(taxcomtab=taxacom6.rmg,tax.select=p.bf.l2$taxuse.rm, showvar="bfNon_exclusiveBF", tax.lev="l2",readjust.p=TRUE,p.adjust.method="fdr",p.cutoff = 1))
```

|  | id | Estimate.bfNon\_exclusiveBF | ll | ul | Pr(>|t|).bfNon\_exclusiveBF | pval.adjust.bfNon\_exclusiveBF |
| --- | --- | --- | --- | --- | --- | --- |
| 1 | k\_\_bacteria.p\_\_actinobacteria | -0.11 | -0.19 | -0.03 | 0.0066 | 0.0266 |
| 5 | k\_\_bacteria.p\_\_proteobacteria | 0.06 | 0.00 | 0.11 | 0.0332 | 0.0665 |
| 2 | k\_\_bacteria.p\_\_bacteroidetes | 0.01 | 0.00 | 0.02 | 0.0580 | 0.0734 |
| 3 | k\_\_bacteria.p\_\_firmicutes | 0.05 | 0.00 | 0.11 | 0.0734 | 0.0734 |

##### Comparison between breastfeeding statuses adjusting for age of infants at sample collection using LM with arcsin squareroot transformation (LMAS)

```
#taxacom6.rmg.as<-taxa.compare(taxtab=taxtab6.rm[[5]],propmed.rel="lm",transform="asin.sqrt",comvar="bf",adjustvar="age.sample",longitudinal="yes",p.adjust.method="fdr")
#phylum
kable(taxcomtab.show(taxcomtab=taxacom6.rmg.as,tax.select=p.bf.l2$taxuse.rm, showvar="bfNon_exclusiveBF", tax.lev="l2",readjust.p=TRUE,p.adjust.method="fdr",p.cutoff = 1))
```

|  | id | Estimate.bfNon\_exclusiveBF | ll | ul | Pr(>|t|).bfNon\_exclusiveBF | pval.adjust.bfNon\_exclusiveBF |
| --- | --- | --- | --- | --- | --- | --- |
| 1 | k\_\_bacteria.p\_\_actinobacteria | -0.13 | -0.23 | -0.03 | 0.0088 | 0.0207 |
| 5 | k\_\_bacteria.p\_\_proteobacteria | 0.10 | 0.02 | 0.17 | 0.0103 | 0.0207 |
| 2 | k\_\_bacteria.p\_\_bacteroidetes | 0.03 | 0.00 | 0.05 | 0.0292 | 0.0390 |
| 3 | k\_\_bacteria.p\_\_firmicutes | 0.07 | 0.00 | 0.14 | 0.0668 | 0.0668 |

#### Example 2: Comparison between infants from 6 months to 2 years of age with solid food introduction after 5 months vs. before 5 months

##### Mean relative abundance by month of solid food introduction and age at phylum, order and family level

```
taxa.meansdn.sl5.rm<-taxa.meansdn(taxtab=taxtab.rm[[5]],sumvar="month.food5",groupvar = "age.sample")
taxa.meansdn.sl5.rm<-taxa.meansdn.sl5.rm[taxa.meansdn.sl5.rm$age.sample>6,]
#phylum
p.sl.l2<-taxa.mean.plot(tabmean=taxa.meansdn.sl5.rm,tax.lev="l2", comvar="month.food5", groupvar="age.sample",mean.filter=0.005, show.taxname="short")
p.sl.l2$p
```

```
#order
p.sl.l4<-taxa.mean.plot(tabmean=taxa.meansdn.sl5.rm,tax.lev="l4", comvar="month.food5", groupvar="age.sample",mean.filter=0.005, show.taxname="short")
p.sl.l4$p
```

```
#family
p.sl.l5<-taxa.mean.plot(tabmean=taxa.meansdn.sl5.rm,tax.lev="l5", comvar="month.food5", groupvar="age.sample",mean.filter=0.005, show.taxname="short")
p.sl.l5$p
```

##### Comparison between infants with solid food introduction after 5 months vs. before 5 months adjusting for age of infants at sample collection using GAMLSS

```
# comparison of bacterial taxa relative abudnance using LMEM or GAMLSS (take some time to run)
#taxacom.6plus.sl5.zi.rmg<-taxa.compare(taxtab=taxtab6plus.rm[[5]],propmed.rel="gamlss",comvar="month.food5",adjustvar="age.sample",longitudinal="yes",p.adjust.method="fdr")
#load saved results
data(taxacom.6plus.sl5.rmg)
#phylum
kable(taxcomtab.show(taxcomtab=taxacom.6plus.sl5.zi.rmg,tax.select=p.sl.l2$taxuse.rm, showvar="food5>5 months", tax.lev="l2",readjust.p=TRUE,p.adjust.method="fdr",p.cutoff = 1))
```

|  | id | Estimate.month.food5>5 months | ll | ul | Pr(>|t|).month.food5>5 months | pval.adjust.food5>5 months |
| --- | --- | --- | --- | --- | --- | --- |
| 2 | k\_\_bacteria.p\_\_bacteroidetes | -0.26 | -0.42 | -0.10 | 0.0018 | 0.0070 |
| 1 | k\_\_bacteria.p\_\_actinobacteria | 0.19 | 0.04 | 0.34 | 0.0119 | 0.0208 |
| 3 | k\_\_bacteria.p\_\_firmicutes | -0.16 | -0.30 | -0.03 | 0.0156 | 0.0208 |
| 5 | k\_\_bacteria.p\_\_proteobacteria | 0.14 | -0.02 | 0.30 | 0.0861 | 0.0861 |

```
#order
kable(taxcomtab.show(taxcomtab=taxacom.6plus.sl5.zi.rmg,tax.select=p.sl.l4$taxuse.rm, showvar="food5>5 months", tax.lev="l4",readjust.p=TRUE,p.adjust.method="fdr",p.cutoff = 1))
```

|  | id | Estimate.month.food5>5 months | ll | ul | Pr(>|t|).month.food5>5 months | pval.adjust.food5>5 months |
| --- | --- | --- | --- | --- | --- | --- |
| 31 | k\_\_bacteria.p\_\_firmicutes.c\_\_clostridia.o\_\_clostridiales | -0.35 | -0.50 | -0.21 | 0.0000 | 0.0000 |
| 26 | k\_\_bacteria.p\_\_bacteroidetes.c\_\_bacteroidia.o\_\_bacteroidales | -0.25 | -0.42 | -0.09 | 0.0022 | 0.0076 |
| 24 | k\_\_bacteria.p\_\_actinobacteria.c\_\_actinobacteria.o\_\_bifidobacteriales | 0.19 | 0.04 | 0.34 | 0.0127 | 0.0297 |
| 29 | k\_\_bacteria.p\_\_firmicutes.c\_\_bacilli.o\_\_lactobacillales | 0.17 | 0.01 | 0.32 | 0.0359 | 0.0628 |
| 39 | k\_\_bacteria.p\_\_proteobacteria.c\_\_gammaproteobacteria.o\_\_enterobacteriales | 0.16 | 0.00 | 0.32 | 0.0543 | 0.0760 |
| 32 | k\_\_bacteria.p\_\_firmicutes.c\_\_erysipelotrichi.o\_\_erysipelotrichales | -0.15 | -0.31 | 0.01 | 0.0662 | 0.0773 |
| 25 | k\_\_bacteria.p\_\_actinobacteria.c\_\_coriobacteriia.o\_\_coriobacteriales | 0.10 | -0.04 | 0.24 | 0.1668 | 0.1668 |

```
#family
kable(taxcomtab.show(taxcomtab=taxacom.6plus.sl5.zi.rmg,tax.select=p.sl.l5$taxuse.rm, showvar="food5>5 months", tax.lev="l5",readjust.p=TRUE,p.adjust.method="fdr",p.cutoff = 1))
```

|  | id | Estimate.month.food5>5 months | ll | ul | Pr(>|t|).month.food5>5 months | pval.adjust.food5>5 months |
| --- | --- | --- | --- | --- | --- | --- |
| 54 | k\_\_bacteria.p\_\_bacteroidetes.c\_\_bacteroidia.o\_\_bacteroidales.f\_\_prevotellaceae | -0.28 | -0.45 | -0.11 | 0.0011 | 0.0107 |
| 73 | k\_\_bacteria.p\_\_firmicutes.c\_\_clostridia.o\_\_clostridiales.f\_\_ruminococcaceae | -0.25 | -0.40 | -0.09 | 0.0016 | 0.0107 |
| 74 | k\_\_bacteria.p\_\_firmicutes.c\_\_clostridia.o\_\_clostridiales.f\_\_veillonellaceae | -0.24 | -0.40 | -0.08 | 0.0034 | 0.0146 |
| 65 | k\_\_bacteria.p\_\_firmicutes.c\_\_bacilli.o\_\_lactobacillales.f\_\_streptococcaceae | 0.23 | 0.07 | 0.39 | 0.0049 | 0.0159 |
| 49 | k\_\_bacteria.p\_\_actinobacteria.c\_\_actinobacteria.o\_\_bifidobacteriales.f\_\_bifidobacteriaceae | 0.19 | 0.04 | 0.34 | 0.0127 | 0.0331 |
| 62 | k\_\_bacteria.p\_\_firmicutes.c\_\_bacilli.o\_\_lactobacillales.f\_\_enterococcaceae | 0.19 | 0.02 | 0.37 | 0.0321 | 0.0619 |
| 71 | k\_\_bacteria.p\_\_firmicutes.c\_\_clostridia.o\_\_clostridiales.f\_\_lachnospiraceae | -0.16 | -0.31 | -0.01 | 0.0339 | 0.0619 |
| 69 | k\_\_bacteria.p\_\_firmicutes.c\_\_clostridia.o\_\_clostridiales.f\_\_clostridiaceae | -0.18 | -0.35 | -0.01 | 0.0381 | 0.0619 |
| 85 | k\_\_bacteria.p\_\_proteobacteria.c\_\_gammaproteobacteria.o\_\_enterobacteriales.f\_\_enterobacteriaceae | 0.16 | 0.00 | 0.32 | 0.0543 | 0.0784 |
| 77 | k\_\_bacteria.p\_\_firmicutes.c\_\_erysipelotrichi.o\_\_erysipelotrichales.f\_\_erysipelotrichaceae | -0.15 | -0.31 | 0.01 | 0.0662 | 0.0861 |
| 50 | k\_\_bacteria.p\_\_actinobacteria.c\_\_coriobacteriia.o\_\_coriobacteriales.f\_\_coriobacteriaceae | 0.10 | -0.04 | 0.24 | 0.1668 | 0.1971 |
| 52 | k\_\_bacteria.p\_\_bacteroidetes.c\_\_bacteroidia.o\_\_bacteroidales.f\_\_bacteroidaceae | -0.01 | -0.19 | 0.17 | 0.8900 | 0.9448 |
| 63 | k\_\_bacteria.p\_\_firmicutes.c\_\_bacilli.o\_\_lactobacillales.f\_\_lactobacillaceae | -0.01 | -0.17 | 0.15 | 0.9448 | 0.9448 |

##### Comparison between infants with solid food introduction after 5 months vs. before 5 months adjusting for age of infants at sample collection using LM

```
#taxacom.6plus.sl5.rmg<-taxa.compare(taxtab=taxtab6plus.rm[[5]],propmed.rel="lm",comvar="month.food5",adjustvar="age.sample",longitudinal="yes",p.adjust.method="fdr")
#phylum
kable(taxcomtab.show(taxcomtab=taxacom.6plus.sl5.rmg,tax.select=p.sl.l2$taxuse.rm, showvar="food5>5 months", tax.lev="l2",readjust.p=TRUE,p.adjust.method="fdr",p.cutoff = 1))
```

|  | id | Estimate.month.food5>5 months | ll | ul | Pr(>|t|).month.food5>5 months | pval.adjust.food5>5 months |
| --- | --- | --- | --- | --- | --- | --- |
| 2 | k\_\_bacteria.p\_\_bacteroidetes | -0.03 | -0.05 | 0.00 | 0.0180 | 0.0721 |
| 3 | k\_\_bacteria.p\_\_firmicutes | -0.04 | -0.11 | 0.04 | 0.3253 | 0.3524 |
| 5 | k\_\_bacteria.p\_\_proteobacteria | 0.01 | -0.01 | 0.04 | 0.3446 | 0.3524 |
| 1 | k\_\_bacteria.p\_\_actinobacteria | 0.04 | -0.05 | 0.14 | 0.3524 | 0.3524 |

```
#order
kable(taxcomtab.show(taxcomtab=taxacom.6plus.sl5.rmg,tax.select=p.sl.l4$taxuse.rm, showvar="food5>5 months", tax.lev="l4",readjust.p=TRUE,p.adjust.method="fdr",p.cutoff = 1))
```

|  | id | Estimate.month.food5>5 months | ll | ul | Pr(>|t|).month.food5>5 months | pval.adjust.food5>5 months |
| --- | --- | --- | --- | --- | --- | --- |
| 31 | k\_\_bacteria.p\_\_firmicutes.c\_\_clostridia.o\_\_clostridiales | -0.06 | -0.11 | -0.02 | 0.0088 | 0.0613 |
| 26 | k\_\_bacteria.p\_\_bacteroidetes.c\_\_bacteroidia.o\_\_bacteroidales | -0.03 | -0.05 | 0.00 | 0.0180 | 0.0631 |
| 32 | k\_\_bacteria.p\_\_firmicutes.c\_\_erysipelotrichi.o\_\_erysipelotrichales | 0.00 | -0.01 | 0.00 | 0.1989 | 0.4640 |
| 39 | k\_\_bacteria.p\_\_proteobacteria.c\_\_gammaproteobacteria.o\_\_enterobacteriales | 0.01 | -0.01 | 0.04 | 0.3020 | 0.4901 |
| 24 | k\_\_bacteria.p\_\_actinobacteria.c\_\_actinobacteria.o\_\_bifidobacteriales | 0.04 | -0.05 | 0.13 | 0.3875 | 0.4901 |
| 29 | k\_\_bacteria.p\_\_firmicutes.c\_\_bacilli.o\_\_lactobacillales | 0.03 | -0.04 | 0.09 | 0.4201 | 0.4901 |
| 25 | k\_\_bacteria.p\_\_actinobacteria.c\_\_coriobacteriia.o\_\_coriobacteriales | 0.00 | -0.01 | 0.02 | 0.5387 | 0.5387 |

```
#family
kable(taxcomtab.show(taxcomtab=taxacom.6plus.sl5.rmg,tax.select=p.sl.l5$taxuse.rm, showvar="food5>5 months", tax.lev="l5",readjust.p=TRUE,p.adjust.method="fdr",p.cutoff = 1))
```

|  | id | Estimate.month.food5>5 months | ll | ul | Pr(>|t|).month.food5>5 months | pval.adjust.food5>5 months |
| --- | --- | --- | --- | --- | --- | --- |
| 54 | k\_\_bacteria.p\_\_bacteroidetes.c\_\_bacteroidia.o\_\_bacteroidales.f\_\_prevotellaceae | -0.03 | -0.05 | -0.01 | 0.0069 | 0.0898 |
| 73 | k\_\_bacteria.p\_\_firmicutes.c\_\_clostridia.o\_\_clostridiales.f\_\_ruminococcaceae | -0.02 | -0.04 | 0.00 | 0.1019 | 0.4487 |
| 65 | k\_\_bacteria.p\_\_firmicutes.c\_\_bacilli.o\_\_lactobacillales.f\_\_streptococcaceae | 0.03 | -0.01 | 0.08 | 0.1484 | 0.4487 |
| 71 | k\_\_bacteria.p\_\_firmicutes.c\_\_clostridia.o\_\_clostridiales.f\_\_lachnospiraceae | -0.02 | -0.05 | 0.01 | 0.1746 | 0.4487 |
| 77 | k\_\_bacteria.p\_\_firmicutes.c\_\_erysipelotrichi.o\_\_erysipelotrichales.f\_\_erysipelotrichaceae | 0.00 | -0.01 | 0.00 | 0.1989 | 0.4487 |
| 52 | k\_\_bacteria.p\_\_bacteroidetes.c\_\_bacteroidia.o\_\_bacteroidales.f\_\_bacteroidaceae | 0.00 | 0.00 | 0.01 | 0.2327 | 0.4487 |
| 74 | k\_\_bacteria.p\_\_firmicutes.c\_\_clostridia.o\_\_clostridiales.f\_\_veillonellaceae | -0.01 | -0.03 | 0.01 | 0.2416 | 0.4487 |
| 85 | k\_\_bacteria.p\_\_proteobacteria.c\_\_gammaproteobacteria.o\_\_enterobacteriales.f\_\_enterobacteriaceae | 0.01 | -0.01 | 0.04 | 0.3020 | 0.4907 |
| 49 | k\_\_bacteria.p\_\_actinobacteria.c\_\_actinobacteria.o\_\_bifidobacteriales.f\_\_bifidobacteriaceae | 0.04 | -0.05 | 0.13 | 0.3875 | 0.5597 |
| 69 | k\_\_bacteria.p\_\_firmicutes.c\_\_clostridia.o\_\_clostridiales.f\_\_clostridiaceae | 0.00 | -0.01 | 0.00 | 0.5061 | 0.6367 |
| 50 | k\_\_bacteria.p\_\_actinobacteria.c\_\_coriobacteriia.o\_\_coriobacteriales.f\_\_coriobacteriaceae | 0.00 | -0.01 | 0.02 | 0.5387 | 0.6367 |
| 62 | k\_\_bacteria.p\_\_firmicutes.c\_\_bacilli.o\_\_lactobacillales.f\_\_enterococcaceae | 0.00 | -0.02 | 0.01 | 0.7033 | 0.7607 |
| 63 | k\_\_bacteria.p\_\_firmicutes.c\_\_bacilli.o\_\_lactobacillales.f\_\_lactobacillaceae | -0.01 | -0.04 | 0.03 | 0.7607 | 0.7607 |

##### Comparison between infants with solid food introduction after 5 months vs. before 5 months adjusting for age of infants at sample collection using LM with arcsin squareroot transformation (LMAS)

```
#taxacom.6plus.sl5.rmg.as<-taxa.compare(taxtab=taxtab6plus.rm[[5]],propmed.rel="lm",transform="asin.sqrt",comvar="month.food5",adjustvar="age.sample",longitudinal="yes",p.adjust.method="fdr")
#phylum
kable(taxcomtab.show(taxcomtab=taxacom.6plus.sl5.rmg.as,tax.select=p.sl.l2$taxuse.rm, showvar="food5>5 months", tax.lev="l2",readjust.p=TRUE,p.adjust.method="fdr",p.cutoff = 1))
```

|  | id | Estimate.month.food5>5 months | ll | ul | Pr(>|t|).month.food5>5 months | pval.adjust.food5>5 months |
| --- | --- | --- | --- | --- | --- | --- |
| 2 | k\_\_bacteria.p\_\_bacteroidetes | -0.05 | -0.09 | -0.01 | 0.0270 | 0.1079 |
| 5 | k\_\_bacteria.p\_\_proteobacteria | 0.02 | -0.02 | 0.07 | 0.2916 | 0.3451 |
| 3 | k\_\_bacteria.p\_\_firmicutes | -0.04 | -0.12 | 0.04 | 0.3168 | 0.3451 |
| 1 | k\_\_bacteria.p\_\_actinobacteria | 0.05 | -0.06 | 0.16 | 0.3451 | 0.3451 |

```
#order
kable(taxcomtab.show(taxcomtab=taxacom.6plus.sl5.rmg.as,tax.select=p.sl.l4$taxuse.rm, showvar="food5>5 months", tax.lev="l4",readjust.p=TRUE,p.adjust.method="fdr",p.cutoff = 1))
```

|  | id | Estimate.month.food5>5 months | ll | ul | Pr(>|t|).month.food5>5 months | pval.adjust.food5>5 months |
| --- | --- | --- | --- | --- | --- | --- |
| 31 | k\_\_bacteria.p\_\_firmicutes.c\_\_clostridia.o\_\_clostridiales | -0.08 | -0.14 | -0.02 | 0.0127 | 0.0887 |
| 26 | k\_\_bacteria.p\_\_bacteroidetes.c\_\_bacteroidia.o\_\_bacteroidales | -0.05 | -0.09 | -0.01 | 0.0268 | 0.0939 |
| 39 | k\_\_bacteria.p\_\_proteobacteria.c\_\_gammaproteobacteria.o\_\_enterobacteriales | 0.03 | -0.02 | 0.08 | 0.2249 | 0.4308 |
| 32 | k\_\_bacteria.p\_\_firmicutes.c\_\_erysipelotrichi.o\_\_erysipelotrichales | -0.01 | -0.04 | 0.01 | 0.3621 | 0.4308 |
| 29 | k\_\_bacteria.p\_\_firmicutes.c\_\_bacilli.o\_\_lactobacillales | 0.04 | -0.05 | 0.12 | 0.3691 | 0.4308 |
| 24 | k\_\_bacteria.p\_\_actinobacteria.c\_\_actinobacteria.o\_\_bifidobacteriales | 0.05 | -0.06 | 0.15 | 0.3693 | 0.4308 |
| 25 | k\_\_bacteria.p\_\_actinobacteria.c\_\_coriobacteriia.o\_\_coriobacteriales | 0.01 | -0.03 | 0.04 | 0.6517 | 0.6517 |

```
#family
kable(taxcomtab.show(taxcomtab=taxacom.6plus.sl5.rmg.as,tax.select=p.sl.l5$taxuse.rm, showvar="food5>5 months", tax.lev="l5",readjust.p=TRUE,p.adjust.method="fdr",p.cutoff = 1))
```

|  | id | Estimate.month.food5>5 months | ll | ul | Pr(>|t|).month.food5>5 months | pval.adjust.food5>5 months |
| --- | --- | --- | --- | --- | --- | --- |
| 54 | k\_\_bacteria.p\_\_bacteroidetes.c\_\_bacteroidia.o\_\_bacteroidales.f\_\_prevotellaceae | -0.06 | -0.10 | -0.01 | 0.0106 | 0.1372 |
| 73 | k\_\_bacteria.p\_\_firmicutes.c\_\_clostridia.o\_\_clostridiales.f\_\_ruminococcaceae | -0.04 | -0.08 | 0.00 | 0.0652 | 0.3699 |
| 65 | k\_\_bacteria.p\_\_firmicutes.c\_\_bacilli.o\_\_lactobacillales.f\_\_streptococcaceae | 0.05 | -0.01 | 0.12 | 0.1139 | 0.3699 |
| 69 | k\_\_bacteria.p\_\_firmicutes.c\_\_clostridia.o\_\_clostridiales.f\_\_clostridiaceae | -0.01 | -0.03 | 0.00 | 0.1404 | 0.3699 |
| 74 | k\_\_bacteria.p\_\_firmicutes.c\_\_clostridia.o\_\_clostridiales.f\_\_veillonellaceae | -0.03 | -0.07 | 0.01 | 0.1423 | 0.3699 |
| 85 | k\_\_bacteria.p\_\_proteobacteria.c\_\_gammaproteobacteria.o\_\_enterobacteriales.f\_\_enterobacteriaceae | 0.03 | -0.02 | 0.08 | 0.2249 | 0.4757 |
| 71 | k\_\_bacteria.p\_\_firmicutes.c\_\_clostridia.o\_\_clostridiales.f\_\_lachnospiraceae | -0.03 | -0.08 | 0.03 | 0.2970 | 0.4757 |
| 62 | k\_\_bacteria.p\_\_firmicutes.c\_\_bacilli.o\_\_lactobacillales.f\_\_enterococcaceae | 0.01 | -0.02 | 0.04 | 0.3343 | 0.4757 |
| 77 | k\_\_bacteria.p\_\_firmicutes.c\_\_erysipelotrichi.o\_\_erysipelotrichales.f\_\_erysipelotrichaceae | -0.01 | -0.04 | 0.01 | 0.3621 | 0.4757 |
| 49 | k\_\_bacteria.p\_\_actinobacteria.c\_\_actinobacteria.o\_\_bifidobacteriales.f\_\_bifidobacteriaceae | 0.05 | -0.06 | 0.15 | 0.3693 | 0.4757 |
| 52 | k\_\_bacteria.p\_\_bacteroidetes.c\_\_bacteroidia.o\_\_bacteroidales.f\_\_bacteroidaceae | 0.01 | -0.01 | 0.03 | 0.4025 | 0.4757 |
| 50 | k\_\_bacteria.p\_\_actinobacteria.c\_\_coriobacteriia.o\_\_coriobacteriales.f\_\_coriobacteriaceae | 0.01 | -0.03 | 0.04 | 0.6517 | 0.7060 |
| 63 | k\_\_bacteria.p\_\_firmicutes.c\_\_bacilli.o\_\_lactobacillales.f\_\_lactobacillaceae | -0.01 | -0.07 | 0.06 | 0.8309 | 0.8309 |

### GAMLSS detects the difference when there is observed difference and does not detect the difference when there is no observed difference

#### Example: Mean bacterial taxa relative abundance in infants from 6 months to 2 years of age by diarrhea status and duration of exclusive breastfeeding

```
taxa.meansdn.dia.exbf2.6plus.rm<-taxa.meansdn(taxtab=taxtab6plus.rm[[5]],sumvar="diarrhea", groupvar="month.exbf2")
#more detail labs
teste<-taxa.meansdn.dia.exbf2.6plus.rm
teste$month.exbf2l<-mapvalues(teste$month.exbf2,from=c("<=2 months",">2 months"),to=c("Duration exbf <=2 months","Duration exbf >2 months"))
#phylum
p.dia.exbf2.6plus.l2<-taxa.mean.plot(tabmean=teste,tax.lev="l2", comvar="diarrhea", groupvar="month.exbf2l",mean.filter=0.005,legend.position="right",ylab="Relative abundance (6 months - 2 years)", show.taxname="short")
p.dia.exbf2.6plus.l2$p
```

#### GAMLSS detects the difference when there are observed difference (In infants with duration of exclusive bf <=2 months)

```
#comparison 

#GAMLSS
#taxacom.6plus.dia.exbf2.zi.rmg<-taxa.compare(taxtab=taxtab6plus.exbf2.rm[[5]],propmed.rel="gamlss",comvar="diarrhea",adjustvar="age.sample",longitudinal="no",p.adjust.method="fdr")
#load saved results 
data(taxacom.dia.exbf2.zi.rmg)
#phylum
kable(taxcomtab.show(taxcomtab=taxacom.6plus.dia.exbf2.zi.rmg,tax.lev="l2",tax.select=p.dia.exbf2.6plus.l2$taxuse.rm,showvar="diarrheaYes",readjust.p=TRUE,p.adjust.method="fdr",p.cutoff = 1))
```

|  | id | Estimate.diarrheaYes | ll | ul | Pr(>|t|).diarrheaYes | pval.adjust.diarrheaYes |
| --- | --- | --- | --- | --- | --- | --- |
| 1 | k\_\_bacteria.p\_\_actinobacteria | -0.73 | -1.12 | -0.34 | 0.0003 | 0.0011 |
| 3 | k\_\_bacteria.p\_\_firmicutes | 0.49 | 0.15 | 0.84 | 0.0055 | 0.0109 |
| 2 | k\_\_bacteria.p\_\_bacteroidetes | -0.29 | -0.68 | 0.10 | 0.1524 | 0.2032 |
| 5 | k\_\_bacteria.p\_\_proteobacteria | -0.17 | -0.54 | 0.20 | 0.3729 | 0.3729 |

```
#LMEM
#taxacom.6plus.dia.exbf2.rmg<-taxa.compare(taxtab=taxtab6plus.exbf2.rm[[5]],propmed.rel="lm",comvar="diarrhea",adjustvar="age.sample",longitudinal="no",p.adjust.method="fdr")
#phylum
kable(taxcomtab.show(taxcomtab=taxacom.6plus.dia.exbf2.rmg,tax.lev="l2",tax.select=p.dia.exbf2.6plus.l2$taxuse.rm,showvar="diarrheaYes",readjust.p=TRUE,p.adjust.method="fdr",p.cutoff=1))
```

|  | id | Estimate.diarrheaYes | ll | ul | Pr(>|t|).diarrheaYes | pval.adjust.diarrheaYes |
| --- | --- | --- | --- | --- | --- | --- |
| 3 | k\_\_bacteria.p\_\_firmicutes | 0.08 | 0.01 | 0.16 | 0.0329 | 0.1317 |
| 1 | k\_\_bacteria.p\_\_actinobacteria | -0.07 | -0.15 | 0.02 | 0.1304 | 0.2608 |
| 2 | k\_\_bacteria.p\_\_bacteroidetes | -0.02 | -0.05 | 0.02 | 0.3068 | 0.4091 |
| 5 | k\_\_bacteria.p\_\_proteobacteria | 0.01 | -0.04 | 0.06 | 0.5909 | 0.5909 |

```
#LMEM with arcsin squareroot transformation
#taxacom.6plus.dia.exbf2.rmg.as<-taxa.compare(taxtab=taxtab6plus.exbf2.rm[[5]],propmed.rel="lm",transform="asin.sqrt",comvar="diarrhea",adjustvar="age.sample",longitudinal="no",p.adjust.method="fdr")
kable(taxcomtab.show(taxcomtab=taxacom.6plus.dia.exbf2.rmg.as,tax.lev="l2",tax.select=p.dia.exbf2.6plus.l2$taxuse.rm,showvar="diarrheaYes",readjust.p=TRUE,p.adjust.method="fdr",p.cutoff=1))
```

|  | id | Estimate.diarrheaYes | ll | ul | Pr(>|t|).diarrheaYes | pval.adjust.diarrheaYes |
| --- | --- | --- | --- | --- | --- | --- |
| 3 | k\_\_bacteria.p\_\_firmicutes | 0.11 | 0.01 | 0.20 | 0.0269 | 0.0848 |
| 1 | k\_\_bacteria.p\_\_actinobacteria | -0.12 | -0.23 | 0.00 | 0.0424 | 0.0848 |
| 2 | k\_\_bacteria.p\_\_bacteroidetes | -0.06 | -0.12 | 0.01 | 0.0852 | 0.1136 |
| 5 | k\_\_bacteria.p\_\_proteobacteria | 0.00 | -0.07 | 0.08 | 0.9060 | 0.9060 |

#### GAMLSS does not detect the difference when there are no observed difference (In infants with duration of exclusive bf >2 months)

```
#GAMLSS
#taxacom.6plus.dia.exbf2plus.zi.rmg<-taxa.compare(taxtab=taxtab6plus.exbf2plus.rm[[5]],propmed.rel="gamlss",comvar="diarrhea",adjustvar="age.sample",longitudinal="no",p.adjust.method="fdr")
#phylum
kable(taxcomtab.show(taxcomtab=taxacom.6plus.dia.exbf2plus.zi.rmg,tax.lev="l2",tax.select=p.dia.exbf2.6plus.l2$taxuse.rm,showvar="diarrheaYes",readjust.p=TRUE,p.adjust.method="fdr",p.cutoff=1))
```

|  | id | Estimate.diarrheaYes | ll | ul | Pr(>|t|).diarrheaYes | pval.adjust.diarrheaYes |
| --- | --- | --- | --- | --- | --- | --- |
| 6 | k\_\_bacteria.p\_\_proteobacteria | 0.12 | -0.33 | 0.56 | 0.6043 | 0.9243 |
| 2 | k\_\_bacteria.p\_\_bacteroidetes | 0.07 | -0.41 | 0.56 | 0.7680 | 0.9243 |
| 4 | k\_\_bacteria.p\_\_firmicutes | -0.02 | -0.40 | 0.36 | 0.9142 | 0.9243 |
| 1 | k\_\_bacteria.p\_\_actinobacteria | 0.02 | -0.42 | 0.46 | 0.9243 | 0.9243 |

```
#LMEM
#taxacom.6plus.dia.exbf2plus.rmg<-taxa.compare(taxtab=taxtab6plus.exbf2plus.rm[[5]],propmed.rel="lm",comvar="diarrhea",adjustvar="age.sample",longitudinal="no",p.adjust.method="fdr")
#phylum
kable(taxcomtab.show(taxcomtab=taxacom.6plus.dia.exbf2plus.rmg,tax.lev="l2",tax.select=p.dia.exbf2.6plus.l2$taxuse.rm,showvar="diarrheaYes",p.adjust.method="fdr",p.cutoff=1))
```

|  | id | Estimate.diarrheaYes | ll | ul | Pr(>|t|).diarrheaYes | pval.adjust.diarrheaYes |
| --- | --- | --- | --- | --- | --- | --- |
| 1 | k\_\_bacteria.p\_\_actinobacteria | 0.02 | -0.08 | 0.12 | 0.6956 | 0.9879 |
| 6 | k\_\_bacteria.p\_\_proteobacteria | -0.01 | -0.06 | 0.05 | 0.7433 | 0.9879 |
| 2 | k\_\_bacteria.p\_\_bacteroidetes | 0.01 | -0.04 | 0.05 | 0.8051 | 0.9879 |
| 4 | k\_\_bacteria.p\_\_firmicutes | -0.01 | -0.10 | 0.08 | 0.8230 | 0.9879 |

```
#LMEM with arcsin squareroot transformation
#taxacom.6plus.dia.exbf2plus.rmg.as<-taxa.compare(taxtab=taxtab6plus.exbf2plus.rm[[5]],propmed.rel="lm",transform="asin.sqrt",comvar="diarrhea",adjustvar="age.sample",longitudinal="no",p.adjust.method="fdr")
#phylum
kable(taxcomtab.show(taxcomtab=taxacom.6plus.dia.exbf2plus.rmg.as,tax.lev="l2",tax.select=p.dia.exbf2.6plus.l2$taxuse.rm,showvar="diarrheaYes",p.adjust.method="fdr",p.cutoff=1))
```

|  | id | Estimate.diarrheaYes | ll | ul | Pr(>|t|).diarrheaYes | pval.adjust.diarrheaYes |
| --- | --- | --- | --- | --- | --- | --- |
| 6 | k\_\_bacteria.p\_\_proteobacteria | 0.02 | -0.06 | 0.11 | 0.5875 | 0.9191 |
| 2 | k\_\_bacteria.p\_\_bacteroidetes | 0.01 | -0.07 | 0.09 | 0.8101 | 0.9707 |
| 1 | k\_\_bacteria.p\_\_actinobacteria | -0.01 | -0.13 | 0.12 | 0.8927 | 0.9707 |
| 4 | k\_\_bacteria.p\_\_firmicutes | 0.00 | -0.10 | 0.10 | 0.9626 | 0.9989 |

## Illustration of meta-analysis workflow

This section illustrate the workflow and examples for the analysis of one microbome study comparing gut microbiome between male vs. female infants <=6 months of age adjusting for feeding status and age at stool sample collection and then meta-analysis across four studies.

### Summary of four included studies

```
#Load example Bangladesh study 
data(sam.rm)
patht<-system.file("extdata/QIIME_outputs/Bangladesh/tax_mapping7", package = "metamicrobiomeR", mustWork = TRUE)
taxrel.rm<-read.multi(patht=patht,patternt=".txt",assignt="no",study="Subramanian et al 2014 (Bangladesh)")
# Bangladesh healthy cohort only and add other meta data
taxrel.ba<-list()
for (j in 1:length(taxrel.rm)){
  taxrel.ba[[j]]<-merge(merge(taxrel.rm[[j]][taxrel.rm[[j]]$x.sampleid %in% samde$fecal.sample.id,],samde, by.x="x.sampleid",by.y="fecal.sample.id"), he50[,c("child.id","gender","month.exbf","month.food")],by.x="personid", by.y="child.id")
}
#all samples from birth to 2 years of age 
nrow(taxrel.ba[[1]])
```

```
[1] 995
```

```
# number of samples for all four studies (for samples <=6 months of age) 
data(studysum)
kable(studysum)
```

|  | female | male | all |
| --- | --- | --- | --- |
| Subramanian et al 2014 (Bangladesh) | 180 | 142 | 322 |
| Bender et al 2016 (Haiti) | 25 | 21 | 46 |
| Pannaraj et al 2017 (USA(CA\_FL)) | 120 | 101 | 221 |
| Thompson et al 2015 (USA(NC)) | 14 | 7 | 21 |
| Sum | 339 | 271 | 610 |

### Comparison of bacterial taxa relative abundance between male vs. female infants < 6 months adjusting for breastfeeding statuses and age of infants at sample collection with GAMLSS

#### Analysis of Bangladesh data

```
# Comparison of bacterial taxa relative abundance up to genus level (take some time to run)
#taxacom6.zi.rm.sex.adjustbfage<-taxa.compare(taxtab=taxtab6.rm[[5]],propmed.rel="gamlss",comvar="gender",adjustvar=c("bf","age.sample"),longitudinal="yes")
# load saved results 
data(taxacom.rm.sex.adjustbfage)
#phylum
kable(taxcomtab.show(taxcomtab=taxacom6.zi.rm.sex.adjustbfage,tax.select="none", showvar="genderMale", tax.lev="l2",p.adjust.method="fdr"))
```

id Estimate.genderMale ll ul Pr(>|t|).genderMale pval.adjust.genderMale — ——————– — — ——————– ———————–

```
#order
kable(taxcomtab.show(taxcomtab=taxacom6.zi.rm.sex.adjustbfage,tax.select="none", showvar="genderMale", tax.lev="l4",p.adjust.method="fdr"))
```

|  | id | Estimate.genderMale | ll | ul | Pr(>|t|).genderMale | pval.adjust.genderMale |
| --- | --- | --- | --- | --- | --- | --- |
| 18 | k\_\_bacteria.p\_\_actinobacteria.c\_\_coriobacteriia.o\_\_coriobacteriales | -0.34 | -0.57 | -0.11 | 0.0040 | 0.1249 |
| 17 | k\_\_bacteria.p\_\_actinobacteria.c\_\_actinobacteria.o\_\_bifidobacteriales | 0.28 | 0.05 | 0.52 | 0.0204 | 0.3061 |

```
#family
kable(taxcomtab.show(taxcomtab=taxacom6.zi.rm.sex.adjustbfage,tax.select="none", showvar="genderMale", tax.lev="l5",p.adjust.method="fdr"))
```

|  | id | Estimate.genderMale | ll | ul | Pr(>|t|).genderMale | pval.adjust.genderMale |
| --- | --- | --- | --- | --- | --- | --- |
| 35 | k\_\_bacteria.p\_\_actinobacteria.c\_\_coriobacteriia.o\_\_coriobacteriales.f\_\_coriobacteriaceae | -0.34 | -0.57 | -0.11 | 0.0040 | 0.1249 |
| 34 | k\_\_bacteria.p\_\_actinobacteria.c\_\_actinobacteria.o\_\_bifidobacteriales.f\_\_bifidobacteriaceae | 0.28 | 0.05 | 0.52 | 0.0204 | 0.3061 |
| 36 | k\_\_bacteria.p\_\_bacteroidetes.c\_\_bacteroidia.o\_\_bacteroidales.f\_\_bacteroidaceae | -0.29 | -0.58 | 0.00 | 0.0491 | 0.4453 |
| 51 | k\_\_bacteria.p\_\_firmicutes.c\_\_clostridia.o\_\_clostridiales.f\_\_eubacteriaceae | -0.98 | -1.95 | -0.01 | 0.0493 | 0.4453 |

```
#genus
kable(taxcomtab.show(taxcomtab=taxacom6.zi.rm.sex.adjustbfage,tax.select="none", showvar="genderMale", tax.lev="l6",p.adjust.method="fdr"))
```

|  | id | Estimate.genderMale | ll | ul | Pr(>|t|).genderMale | pval.adjust.genderMale |
| --- | --- | --- | --- | --- | --- | --- |
| 114 | k\_\_bacteria.p\_\_firmicutes.c\_\_erysipelotrichi.o\_\_erysipelotrichales.f\_\_erysipelotrichaceae.g\_\_.eubacterium. | -0.70 | -1.15 | -0.25 | 0.0024 | 0.1249 |
| 72 | k\_\_bacteria.p\_\_actinobacteria.c\_\_coriobacteriia.o\_\_coriobacteriales.f\_\_coriobacteriaceae.g\_\_collinsella | -0.35 | -0.63 | -0.06 | 0.0165 | 0.3061 |
| 69 | k\_\_bacteria.p\_\_actinobacteria.c\_\_actinobacteria.o\_\_bifidobacteriales.f\_\_bifidobacteriaceae.g\_\_bifidobacterium | 0.28 | 0.05 | 0.52 | 0.0204 | 0.3061 |
| 99 | k\_\_bacteria.p\_\_firmicutes.c\_\_clostridia.o\_\_clostridiales.f\_\_lachnospiraceae.g\_\_.ruminococcus. | 0.42 | 0.06 | 0.79 | 0.0222 | 0.3061 |
| 74 | k\_\_bacteria.p\_\_bacteroidetes.c\_\_bacteroidia.o\_\_bacteroidales.f\_\_bacteroidaceae.g\_\_bacteroides | -0.29 | -0.58 | 0.00 | 0.0491 | 0.4453 |
| 93 | k\_\_bacteria.p\_\_firmicutes.c\_\_clostridia.o\_\_clostridiales.f\_\_eubacteriaceae.g\_\_pseudoramibacter\_eubacterium | -0.98 | -1.95 | -0.01 | 0.0493 | 0.4453 |

The analysis for other studies was done similarly.

#### Meta-analysis of four studies (Bangladesh, Haiti, USA(CA\_FL), USA(NC))

Heatmap of log(odds ratio) (log(OR)) of relative abundances of gut bacterial taxa at different taxonomic levels between male vs. female infants for each study and pooled estimates (meta-analysis) across all studies with 95% confidence intervals (95% CI) (forest plot). All log(OR) estimates of each bacterial taxa from each study were from Generalized Additive Models for Location Scale and Shape (GAMLSS) with beta zero inflated family (BEZI) and were adjusted for feeding status and age of infants at sample collection. Pooled log(OR) estimates and 95% CI (forest plot) were from random effect meta-analysis models with inverse variance weighting and DerSimonian-Laird estimator for between-study variance based on the adjusted log(OR) estimates and corresponding standard errors of all included studies. Bacterial taxa with p-values for differential relative abundances <0.05 were denoted with \* and those with p-values <0.0001 were denoted with \*\*. Pooled log(OR) estimates with pooled p-values<0.05 are in red and those with false discovery rate (FDR) adjusted pooled p-values <0.1 are in triangle shape. Missing (unavailable) values are in white. USA: United States of America; CA: California; FL: Florida; NC: North Carolina.

```
# load saved results of four studies for the comparison of bacterial taxa relative abundance between genders adjusted for breastfeeding and infant age at sample collection 
data(taxacom.rm.sex.adjustbfage)
data(taxacom.ha.sex.adjustbfage)
data(taxacom6.zi.usbmk.sex.adjustbfage)
data(taxacom6.unc.sex.adjustedbfage)
taxacom6.zi.rm.sex.adjustbfage$study<-"Subramanian et al 2014 (Bangladesh)"
taxacom6.zi.rm.sex.adjustbfage$pop<-"Bangladesh"
taxacom.zi.ha.sex.adjustbfage$study<-"Bender et al 2016 (Haiti)"
taxacom.zi.ha.sex.adjustbfage$pop<-"Haiti"
taxacom6.zi.usbmk.sex.adjustbfage$study<-"Pannaraj et al 2017 (USA(CA_FL))"
taxacom6.zi.usbmk.sex.adjustbfage$pop<-"USA(CA_FL)"
taxacom6.zi.unc.sex.adjustedbfage$study<-"Thompson et al 2015 (USA(NC))"
taxacom6.zi.unc.sex.adjustedbfage$pop<-"USA(NC)"
tabsex4<-rbind.fill(taxacom6.zi.rm.sex.adjustbfage,taxacom.zi.ha.sex.adjustbfage,taxacom6.zi.usbmk.sex.adjustbfage,taxacom6.zi.unc.sex.adjustedbfage)
# meta-analysis (take some time to run)
#metab.sex<-meta.taxa(taxcomdat=tabsex4,summary.measure="RR",pool.var="id",studylab="study",backtransform=FALSE,percent.meta=0.5,p.adjust.method="fdr")
#load saved results 
data(metab.sex)
#phylum
kable(metatab.show(metatab=metab.sex$random,com.pooled.tab=tabsex4,tax.lev="l2",showvar="genderMale",p.cutoff.type="p", p.cutoff=0.05,display="table"))
```

id estimate ll ul p p.adjust — ——— — — — ———

```
#plot
metadat<-metatab.show(metatab=metab.sex$random,com.pooled.tab=tabsex4,tax.lev="l2",showvar="genderMale",p.cutoff.type="p", p.cutoff=1,display="data")
meta.niceplot(metadat=metadat,sumtype="taxa",level="main",p="p",p.adjust="p.adjust",phyla.col="rainbow",p.sig.heat="yes",heat.forest.width.ratio =c(1.5,1),leg.key.size=0.8,leg.text.size=10,heat.text.x.size=10,heat.text.x.angle=0,forest.axis.text.y=8,forest.axis.text.x=10, point.ratio = c(4,2),line.ratio = c(2,1))
```

```
#order 
kable(metatab.show(metatab=metab.sex$random,com.pooled.tab=tabsex4,tax.lev="l4",showvar="genderMale",p.cutoff.type="p", p.cutoff=0.05,display="table"))
```

|  | id | estimate | ll | ul | p | p.adjust |
| --- | --- | --- | --- | --- | --- | --- |
| 18 | k\_\_bacteria.p\_\_actinobacteria.c\_\_coriobacteriia.o\_\_coriobacteriales | -0.26 | -0.44 | -0.08 | 0.0049 | 0.209 |

```
#some different plot options: increase size of forest plot vs. heatmap, change color palette, legend size 
metadat<-metatab.show(metatab=metab.sex$random,com.pooled.tab=tabsex4,tax.lev="l4",showvar="genderMale",p.cutoff.type="p", p.cutoff=1,display="data")
meta.niceplot(metadat=metadat,sumtype="taxa",level="sub",p="p",p.adjust="p.adjust",phyla.col="rainbow",leg.key.size=1,leg.text.size=8,heat.text.x.size=6,forest.axis.text.y=8,forest.axis.text.x=6,heat.forest.width.ratio =c(1,1.3), neg.palette = "Greens",pos.palette = "Purples", point.ratio = c(4,2),line.ratio = c(2,1))
```

```
# family 
kable(metatab.show(metatab=metab.sex$random,com.pooled.tab=tabsex4,tax.lev="l5",showvar="genderMale",p.cutoff.type="p", p.cutoff=0.05,display="table"))
```

|  | id | estimate | ll | ul | p | p.adjust |
| --- | --- | --- | --- | --- | --- | --- |
| 35 | k\_\_bacteria.p\_\_actinobacteria.c\_\_coriobacteriia.o\_\_coriobacteriales.f\_\_coriobacteriaceae | -0.26 | -0.44 | -0.08 | 0.0049 | 0.2090 |
| 50 | k\_\_bacteria.p\_\_firmicutes.c\_\_clostridia.o\_\_clostridiales.f\_\_eubacteriaceae | -0.68 | -1.35 | -0.02 | 0.0436 | 0.9833 |

```
#(not show significant p-values of each study in heatmap)
metadat<-metatab.show(metatab=metab.sex$random,com.pooled.tab=tabsex4,tax.lev="l5",showvar="genderMale",p.cutoff.type="p", p.cutoff=1,display="data")
meta.niceplot(metadat=metadat,sumtype="taxa",level="sub",p="p",p.adjust="p.adjust",phyla.col="rainbow",leg.key.size=1,p.sig.heat ="no",leg.text.size=8,heat.text.x.size=7,forest.axis.text.y=8,forest.axis.text.x=7, point.ratio = c(4,2),line.ratio = c(2,1))
```

```
#genus 
kable(metatab.show(metatab=metab.sex$random,com.pooled.tab=tabsex4,tax.lev="l6",showvar="genderMale",p.cutoff.type="p", p.cutoff=0.05,display="table"))
```

|  | id | estimate | ll | ul | p | p.adjust |
| --- | --- | --- | --- | --- | --- | --- |
| 165 | k\_\_bacteria.p\_\_firmicutes.c\_\_clostridia.o\_\_clostridiales.f\_\_lachnospiraceae.g\_\_coprococcus | 0.43 | 0.42 | 0.44 | 0.0000 | 0.0000 |
| 108 | k\_\_bacteria.p\_\_firmicutes.c\_\_erysipelotrichi.o\_\_erysipelotrichales.f\_\_erysipelotrichaceae.g\_\_.eubacterium. | -0.44 | -0.76 | -0.13 | 0.0056 | 0.2090 |
| 100 | k\_\_bacteria.p\_\_firmicutes.c\_\_clostridia.o\_\_clostridiales.f\_\_veillonellaceae.g\_\_megamonas | -0.47 | -0.91 | -0.02 | 0.0410 | 0.9833 |
| 87 | k\_\_bacteria.p\_\_firmicutes.c\_\_clostridia.o\_\_clostridiales.f\_\_eubacteriaceae.g\_\_pseudoramibacter\_eubacterium | -0.68 | -1.35 | -0.02 | 0.0436 | 0.9833 |

```
#some different plot options: pooled estimates in forest plot with the same color scales as heatmap, those with p-values<0.05 in bold, FDR adjusted p-values<0.1 in triangles
metadat<-metatab.show(metatab=metab.sex$random,com.pooled.tab=tabsex4,tax.lev="l6",showvar="genderMale",p.cutoff.type="p", p.cutoff=1,display="data")
meta.niceplot(metadat=metadat,sumtype="taxa",level="sub",p="p",p.adjust="p.adjust",phyla.col="rainbow",p.sig.heat="yes",heat.forest.width.ratio =c(1,1.3),forest.col="by.estimate",leg.key.size=0.8,leg.text.size=10,heat.text.x.size=6,forest.axis.text.y=7,forest.axis.text.x=6, point.ratio = c(4,2),line.ratio = c(2,1))
```

### Comparison of relative abundance of bacterial functional (KEGG) pathways between male vs. female infants < 6 months adjusting for breastfeeding statuses and age of infants at sample collection

#### Analysis for Bangladesh study

```
# RM
data(sam.rm)
patht<-system.file("extdata/QIIME_outputs/Bangladesh/picrust", package = "metamicrobiomeR", mustWork = TRUE)
kegg<-read.multi(patht=patht,patternt=".txt",assignt="no")
kegg.rm<-list()
for (i in 1:length(kegg)){
  rownames(kegg[[i]])<-kegg[[i]][,"kegg_pathways"]
  kegg[[i]]<-kegg[[i]][,colnames(kegg[[i]])[!colnames(kegg[[i]]) %in% c("otu.id","kegg_pathways")]]
  kegg.rm[[i]]<-as.data.frame(t(kegg[[i]]))
}
covar.rm<-merge(samde, he50[,c("child.id","gender","zygosity","day.firstsample","day.lastsample","n.sample","sampling.interval.msd","month.exbf","month.food",
                               "n.diarrhea.yr","percent.time.diarrhea","fraction.antibiotic","subject.allocation")], by="child.id")
covar.rm<-dplyr::rename(covar.rm,sampleid=fecal.sample.id, personid=child.id ,age.sample=age.months)
covar.rm$bf<-factor(covar.rm$bf, levels=c('ExclusiveBF','Non_exclusiveBF','No_BF'))

covar.rm$personid<-as.factor(covar.rm$personid)
# Comparison of pathway relative abundances (take time to run)
#pathcom.rm6.rel.gamlss.sexg<-pathway.compare(pathtab=kegg.rm,mapfile=covar.rm,sampleid="sampleid",pathsum="rel",stat.med="gamlss",comvar="gender",adjustvar=c("age.sample","bf"),longitudinal="yes",p.adjust.method="fdr",percent.filter=0.05,relabund.filter=0.00005,age.limit=6)
# load saved results 
data(pathcom.rm6.rel.gamlss.sexg)
kable(taxcomtab.show(taxcomtab=pathcom.rm6.rel.gamlss.sexg$l2, sumvar="path",tax.lev="l2",tax.select="none",showvar="genderMale", p.adjust.method="fdr",p.cutoff=0.05))
```

|  | id | Estimate.genderMale | ll | ul | Pr(>|t|).genderMale | pval.adjust.genderMale |
| --- | --- | --- | --- | --- | --- | --- |
| 1 | Metabolism..Amino.Acid.Metabolism | 0.02 | 0.00 | 0.04 | 0.0302 | 0.2739 |
| 14 | Genetic.Information.Processing..Folding..Sorting.and.Degradation | -0.01 | -0.01 | 0.00 | 0.0331 | 0.2739 |
| 9 | Organismal.Systems..Endocrine.System | 0.07 | 0.00 | 0.13 | 0.0391 | 0.2739 |
| 19 | Human.Diseases..Infectious.Diseases | -0.03 | -0.06 | 0.00 | 0.0404 | 0.2739 |
| 2 | Metabolism..Biosynthesis.of.Other.Secondary.Metabolites | 0.03 | 0.00 | 0.06 | 0.0413 | 0.2739 |

```
kable(taxcomtab.show(taxcomtab=pathcom.rm6.rel.gamlss.sexg$l3, sumvar="path",tax.lev="l3",tax.select="none",showvar="genderMale", p.adjust.method="fdr",p.cutoff=0.05))
```

|  | id | Estimate.genderMale | ll | ul | Pr(>|t|).genderMale | pval.adjust.genderMale |
| --- | --- | --- | --- | --- | --- | --- |
| 15 | Metabolism..Amino.Acid.Metabolism..Arginine.and.proline.metabolism | 0.02 | 0.01 | 0.04 | 0.0055 | 0.565 |
| 44 | Genetic.Information.Processing..Folding..Sorting.and.Degradation..Chaperones.and.folding.catalysts | -0.02 | -0.04 | -0.01 | 0.0085 | 0.565 |
| 139 | Metabolism..Amino.Acid.Metabolism..Phenylalanine..tyrosine.and.tryptophan.biosynthesis | 0.05 | 0.01 | 0.08 | 0.0112 | 0.565 |
| 140 | Metabolism..Biosynthesis.of.Other.Secondary.Metabolites..Phenylpropanoid.biosynthesis | 0.09 | 0.02 | 0.16 | 0.0159 | 0.565 |
| 33 | Metabolism..Carbohydrate.Metabolism..C5.Branched.dibasic.acid.metabolism | 0.05 | 0.01 | 0.08 | 0.0168 | 0.565 |
| 38 | Metabolism..Energy.Metabolism..Carbon.fixation.pathways.in.prokaryotes | -0.02 | -0.03 | 0.00 | 0.0251 | 0.565 |
| 123 | Metabolism..Glycan.Biosynthesis.and.Metabolism..Other.glycan.degradation | 0.11 | 0.01 | 0.20 | 0.0257 | 0.565 |
| 21 | Environmental.Information.Processing..Signaling.Molecules.and.Interaction..Bacterial.toxins | 0.09 | 0.01 | 0.17 | 0.0257 | 0.565 |
| 203 | Environmental.Information.Processing..Membrane.Transport..Transporters | 0.03 | 0.00 | 0.05 | 0.0293 | 0.565 |
| 45 | Metabolism..Xenobiotics.Biodegradation.and.Metabolism..Chloroalkane.and.chloroalkene.degradation | 0.04 | 0.00 | 0.08 | 0.0309 | 0.565 |
| 128 | Organismal.Systems..Endocrine.System..PPAR.signaling.pathway | 0.08 | 0.01 | 0.16 | 0.0348 | 0.565 |
| 80 | Metabolism..Lipid.Metabolism..Glycerophospholipid.metabolism | -0.03 | -0.06 | 0.00 | 0.0356 | 0.565 |
| 81 | Metabolism..Amino.Acid.Metabolism..Glycine..serine.and.threonine.metabolism | 0.02 | 0.00 | 0.03 | 0.0362 | 0.565 |
| 213 | Metabolism..Amino.Acid.Metabolism..Valine..leucine.and.isoleucine.biosynthesis | 0.03 | 0.00 | 0.06 | 0.0383 | 0.565 |
| 2 | Organismal.Systems..Endocrine.System..Adipocytokine.signaling.pathway | 0.10 | 0.00 | 0.19 | 0.0427 | 0.565 |
| 50 | Metabolism..Amino.Acid.Metabolism..Cysteine.and.methionine.metabolism | 0.02 | 0.00 | 0.05 | 0.0461 | 0.565 |
| 182 | Metabolism..Metabolism.of.Other.Amino.Acids..Selenocompound.metabolism | 0.02 | 0.00 | 0.04 | 0.0463 | 0.565 |

The analyses for data of other studies were done similarly.

#### Meta-analysis of four studies

```
#load save results of four studies for the comparison of pathway relative abundance between genders adjusted for breastfeeding status and infant age at sample collection 
data(pathcom.unc6.rel.gamlss.sexg)
data(pathcom.ha6.rel.gamlss.sexg)
data(pathcom.rm6.rel.gamlss.sexg)
data(pathcom.usbmk6.rel.gamlss.sexg)
#Bangladesh
taxacom.zi.rm<-pathcom.rm6.rel.gamlss.sexg
for (i in 1: length(names(taxacom.zi.rm))){
  taxacom.zi.rm[[i]]<-as.data.frame(taxacom.zi.rm[[i]])
  taxacom.zi.rm[[i]][,'path']<-rownames(taxacom.zi.rm[[i]])
  taxacom.zi.rm[[i]][,'study']<-"Subramanian et al 2014 (Bangladesh)"
  taxacom.zi.rm[[i]][,'pop']<-"Bangladesh"
}
#Haiti
taxacom.zi.ha<-pathcom.ha6.rel.gamlss.sexg
for (i in 1: length(names(taxacom.zi.ha))){
  taxacom.zi.ha[[i]]<-as.data.frame(taxacom.zi.ha[[i]])
  taxacom.zi.ha[[i]][,'path']<-rownames(taxacom.zi.ha[[i]])
  taxacom.zi.ha[[i]][,'study']<-"Bender et al 2016 (Haiti)"
  taxacom.zi.ha[[i]][,'pop']<-"Haiti"
}
#CA-FL
taxacom.zi.usbmk<-pathcom.usbmk6.rel.gamlss.sexg
for (i in 1: length(names(taxacom.zi.usbmk))){
  taxacom.zi.usbmk[[i]]<-as.data.frame(taxacom.zi.usbmk[[i]])
  taxacom.zi.usbmk[[i]][,'path']<-rownames(taxacom.zi.usbmk[[i]])
  taxacom.zi.usbmk[[i]][,'study']<-"Pannaraj et al 2017 (USA(CA_FL))"
  taxacom.zi.usbmk[[i]][,'pop']<-"USA(CA_FL)"
}
#NC
taxacom.zi.unc<-pathcom.unc6.rel.gamlss.sexg
for (i in 1:length(names(taxacom.zi.unc))){ #
  taxacom.zi.unc[[i]]<-as.data.frame(taxacom.zi.unc[[i]])
  taxacom.zi.unc[[i]][,'path']<-rownames(taxacom.zi.unc[[i]])
  taxacom.zi.unc[[i]][,'study']<-"Thompson et al 2015 (USA(NC))"
  taxacom.zi.unc[[i]][,'pop']<-"USA(NC)"
}

taxacom.zi.l2<-rbind.fill(taxacom.zi.rm$l2,taxacom.zi.ha$l2,taxacom.zi.unc$l2,taxacom.zi.usbmk$l2)
#taxacom.zi.l2$pop<-as.factor(taxacom.zi.l2$pop)
taxacom.zi.l3<-rbind.fill(taxacom.zi.rm$l3,taxacom.zi.ha$l3,taxacom.zi.unc$l3,taxacom.zi.usbmk$l3)
#taxacom.zi.l3$pop<-as.factor(taxacom.zi.l3$pop)
pathcom.zi.sexg<-list(l2=taxacom.zi.l2,l3=taxacom.zi.l3)

# meta-analysis (take some time to run)
#pathmetatab.zi.sex.l2<-meta.taxa(taxcomdat=pathcom.zi.sexg$l2, sm="RR",studylab = "pop", p.adjust.method="fdr",percent.meta=0.5,pool.var="id")
#pathmetatab.zi.sex.l3<-meta.taxa(taxcomdat=pathcom.zi.sexg$l3, sm="RR",studylab = "pop", p.adjust.method="fdr",percent.meta=0.5,pool.var="id")
# load saved results 
data(pathmetatab.zi.sexg) 

#level 2
kable(metatab.show(metatab=pathmetatab.zi.sex.l2$random,com.pooled.tab=pathcom.zi.sexg$l2,sumvar="path",showvar="genderMale",p.cutoff.type="p", p.cutoff=0.05,display="table"))
```

|  | id | estimate | ll | ul | p | p.adjust |
| --- | --- | --- | --- | --- | --- | --- |
| 26 | Metabolism..Metabolism.of.Terpenoids.and.Polyketides | -0.01 | -0.02 | 0 | 0.0417 | 0.9902 |

```
# Nice plot all pathways (use different color scale for pathway)
metadat<-metatab.show(metatab=pathmetatab.zi.sex.l2$random,com.pooled.tab=pathcom.zi.sexg$l2,sumvar="path",showvar="genderMale",p.cutoff.type="p", p.cutoff=1,display="data")
metadat$taxsig.all$pop<-factor(metadat$taxsig.all$pop,levels=c("Bangladesh","Haiti","USA(CA_FL)","USA(NC)","Pooled"))
meta.niceplot(metadat=metadat,sumtype="path",p="p",p.adjust="p.adjust",p.sig.heat="yes",heat.forest.width.ratio =c(1,1.3),est.break = c(-Inf, -0.5,-0.1,-0.05,0,0.05,0.1,0.5, Inf),est.break.label = c("<-0.5)", "[-0.5,-0.1)","[-0.1,-0.05)","[-0.05,0)","[0,0.05)","[0.05,0.1)", "[0.1,0.5)",">=0.5"),leg.key.size=0.8,leg.text.size=10,heat.text.x.size=6,forest.axis.text.y=6,forest.axis.text.x=6, point.ratio = c(4,2),line.ratio = c(2,1))
```

```
#Level 3
kable(metatab.show(metatab=pathmetatab.zi.sex.l3$random,com.pooled.tab=pathcom.zi.sexg$l3,sumvar="path",showvar="genderMale",p.cutoff.type="p", p.cutoff=0.05,display="table"))
```

|  | id | estimate | ll | ul | p | p.adjust |
| --- | --- | --- | --- | --- | --- | --- |
| 40 | Unclassified..Cellular.Processes.and.Signaling..Cell.division | 0.08 | 0.01 | 0.16 | 0.0324 | 0.9975 |
| 98 | Metabolism..Lipid.Metabolism..Lipid.biosynthesis.proteins | -0.01 | -0.02 | 0.00 | 0.0411 | 0.9975 |
| 16 | Metabolism..Carbohydrate.Metabolism..Ascorbate.and.aldarate.metabolism | 0.03 | 0.00 | 0.06 | 0.0472 | 0.9975 |

```
# Nice plot for pathways with pooled p-values<=0.3
metadat<-metatab.show(metatab=pathmetatab.zi.sex.l3$random,com.pooled.tab=pathcom.zi.sexg$l3,sumvar="path",showvar="genderMale",p.cutoff.type="p", p.cutoff=0.3,display="data")
metadat$taxsig.all$pop<-factor(metadat$taxsig.all$pop,levels=c("Bangladesh","Haiti","USA(CA_FL)","USA(NC)","Pooled"))
meta.niceplot(metadat=metadat,sumtype="path",p="p",p.adjust="p.adjust",est.break = c(-Inf, -0.5,-0.1,-0.05,0,0.05,0.1,0.5, Inf),est.break.label = c("<-0.5)", "[-0.5,-0.1)","[-0.1,-0.05)","[-0.05,0)","[0,0.05)","[0.05,0.1)","[0.1,0.5)",">=0.5"),heat.forest.width.ratio=c(1,1.3),leg.key.size=1,leg.text.size=8,heat.text.x.size=6,forest.axis.text.y=6,forest.axis.text.x=6, point.ratio = c(4,2),line.ratio = c(2,1))
```

### Meta-analysis of other microbiome measures

Random effects meta-analysis models can also be generally applied to other microbiome measures such as microbial alpha diversity and microbiome age. To make the estimates for these positive continuous microbiome measures comparable across studies, these measures should be standardized to have a mean of 0 and standard deviation of 1 before between-group-comparison within each study. Random effects meta-analysis models can then be applied to pool the “comparable” estimates and their standard errors across studies. Meta-analysis results of these measures can be displayed as standard meta-analysis forest plots.

#### Alpha diversity

##### Calculate mean alpha diversity indexes for a selected rarefaction depth, standardize and compare standardized alpha diversity indexes between groups (male vs. female infants <=6 months of age) adjusting for covariates (feeding status and infant age at sample collection) using Bangladesh data

For each study, the *alpha.compare* function imports the outputs from “alpha\_rarefaction.py” QIIME1 script and calculates mean alpha diversity for different indices for each sample based on a user defined rarefaction depth. Mean alpha diversity indexes are standardized to have a mean of 0 and standard deviation of 1 to make these measures comparable across studies. Standardized alpha diversity indexes are compared between groups adjusting for covariates using LM. Meta-analysis across studies is then done and the results are displayed as a standard meta-analysis forest plot.

```
data(sam.rm)
patht<-system.file("extdata/QIIME_outputs/Bangladesh/alpha_div_collated", package = "metamicrobiomeR", mustWork = TRUE)
alpha.rm<-read.multi(patht=patht,patternt=".txt",assignt="no",study="Bangladesh")
names(alpha.rm)<-sub(patht,"",names(alpha.rm))
samfile<-merge(samde, he50[,c("child.id","gender","month.exbf","month.food")],by="child.id")
samfile$age.sample<-samfile$age.months
samfile$bf<-factor(samfile$bf,levels=c("ExclusiveBF","Non_exclusiveBF","No_BF"))
samfile$personid<-samfile$child.id
samfile$sampleid<-tolower(samfile$fecal.sample.id)
#comparison of standardized alpha diversity indexes between genders adjusting for breastfeeding and infant age at sample collection in infants <=6 months of age 
alphacom6.rm.sexsg<-alpha.compare(datlist=alpha.rm,depth=3,mapfile=samfile,mapsampleid="fecal.sample.id",comvar="gender",adjustvar=c("age.sample","bf"),longitudinal="yes",age.limit=6,standardize=TRUE)
kable(alphacom6.rm.sexsg$alphasum[,1:5])
```

| id | Estimate.genderMale | Std. Error.genderMale | t value.genderMale | Pr(>|t|).genderMale |
| --- | --- | --- | --- | --- |
| chao1 | 0.0652722 | 0.0721873 | 0.9042059 | 0.3658862 |
| observed\_species | 0.0652941 | 0.0621281 | 1.0509593 | 0.2932773 |
| pd\_whole\_tree | 0.0313157 | 0.0502442 | 0.6232707 | 0.5331066 |
| shannon | -0.0012757 | 0.0820824 | -0.0155418 | 0.9875999 |

```
alpha.sexs<-merge(samfile,alphacom6.rm.sexsg$alphamean.standardized,by="sampleid")

#plot curves of standardized Shannon index by age and gender with Generalized Additive Mixed Models (GAMM) 
alpha.sexs$gender<-as.factor(alpha.sexs$gender)
alpha.sexs$bf<-as.factor(alpha.sexs$bf)
gfit<-gamm(shannon~s(age.sample,by=gender) +gender,family=gaussian,
         data=alpha.sexs,random=list(personid=~1))
pred <- predict(gfit$gam, newdata = alpha.sexs,se.fit=TRUE)
datfit<-cbind(alpha.sexs, fit=pred$fit,ul=(pred$fit+(1.96*pred$se.fit)),ll=(pred$fit-(1.96*pred$se.fit)))
ggplot()+ geom_point(data = subset(alpha.sexs,age.sample<=6), aes(x = age.sample, y = shannon, group = personid, colour=gender),size=1)+ 
  geom_line(data = subset(alpha.sexs,age.sample<=6), aes(x = age.sample, y = shannon, group = personid, colour=gender),size=0.1)+
  geom_line(data = subset(datfit,age.sample<=6),aes(x = age.sample, y = fit, colour=gender),size = 1)+
  geom_ribbon(data = subset(datfit,age.sample<=6),aes(x=age.sample, ymax=ul, ymin=ll, fill=gender), alpha=.5)+guides(fill=FALSE)+
  xlab("Chronological age (month)") +ylab("Standardized Shannon index")+
  scale_x_continuous(breaks=seq(from=0,to=24,by=3),
                     labels=seq(from=0,to=24,by=3))+
  labs(color='')+
  theme(legend.position = "right",
        axis.line = element_line(colour = "black"),
        panel.grid.major = element_blank(),
        panel.grid.minor = element_blank(),
        panel.border = element_blank(),
        panel.background = element_blank(),
        strip.background =element_rect(fill="white"))
```

The analyses for other studies were done similarly.

#### Meta-analysis of four studies

The results showed that alpha diversity (four commonly used indexes Shannon, Phylogenetic diversity whole tree, Observed species, Chao1) was not different between male and female infants <=6 months of age in the meta-analysis of the four included studies.

```
# load saved results of 4 studies 
data(alphacom6.sex4.scaledg)
# put data from 4 studies together for meta-analysis 
asum.ba<-alphacom6.rm.sexsg$alphasum
asum.ba$pop<-"Bangladesh"
asum.ha<-alphacom6.ha.sexsg$alphasum
asum.ha$pop<-"Haiti"
asum.cafl<-alphacom6.usbmk.sexsg$alphasum
asum.cafl$pop<-"USA(CA_FL)"
asum.unc<-alphacom6.unc.sexsg$alphasum
asum.unc$pop<-"USA(UNC)"
asum4<-rbind.fill(asum.ba,asum.ha,asum.cafl,asum.unc)
kable(asum4[,c(colnames(asum4)[1:5],"pop")])
```

| id | Estimate.genderMale | Std. Error.genderMale | t value.genderMale | Pr(>|t|).genderMale | pop |
| --- | --- | --- | --- | --- | --- |
| chao1 | 0.0652722 | 0.0721873 | 0.9042059 | 0.3658862 | Bangladesh |
| observed\_species | 0.0652941 | 0.0621281 | 1.0509600 | 0.2932770 | Bangladesh |
| pd\_whole\_tree | 0.0313157 | 0.0502441 | 0.6232717 | 0.5331060 | Bangladesh |
| shannon | -0.0012757 | 0.0820824 | -0.0155418 | 0.9875999 | Bangladesh |
| chao1 | -0.0886223 | 0.3528103 | -0.2511895 | 0.8029223 | Haiti |
| observed\_species | -0.0914700 | 0.3453005 | -0.2648999 | 0.7924139 | Haiti |
| pd\_whole\_tree | 0.0425683 | 0.2952372 | 0.1441835 | 0.8860620 | Haiti |
| shannon | -0.0091118 | 0.2935656 | -0.0310382 | 0.9753897 | Haiti |
| chao1 | -0.0450324 | 0.1337008 | -0.3368147 | 0.7362566 | USA(CA\_FL) |
| observed\_species | -0.0653818 | 0.1144946 | -0.5710474 | 0.5679675 | USA(CA\_FL) |
| pd\_whole\_tree | -0.1590197 | 0.1075934 | -1.4779691 | 0.1394160 | USA(CA\_FL) |
| shannon | -0.0705346 | 0.1433712 | -0.4919718 | 0.6227392 | USA(CA\_FL) |
| chao1 | 0.4965750 | 0.5924682 | 0.8381463 | 0.4019485 | USA(UNC) |
| observed\_species | 0.2336717 | 0.5294163 | 0.4413761 | 0.6589407 | USA(UNC) |
| pd\_whole\_tree | -0.0107622 | 0.7331424 | -0.0146796 | 0.9882878 | USA(UNC) |
| shannon | 0.1783060 | 0.5552779 | 0.3211113 | 0.7481260 | USA(UNC) |

```
#Shannon index 
shannon.sex <- metagen(Estimate.genderMale, `Std. Error.genderMale`, studlab=pop,data=subset(asum4,id=="shannon"),sm="RD", backtransf=FALSE)
forest(shannon.sex,smlab="Standardized \n diversity difference",sortvar=subset(asum4,id=="shannon")$pop,lwd=2)
```

```
shannon.sex
```

```
                RD            95%-CI %W(fixed) %W(random)
Bangladesh -0.0013 [-0.1622; 0.1596]      70.0       70.0
Haiti      -0.0091 [-0.5845; 0.5663]       5.5        5.5
USA(CA_FL) -0.0705 [-0.3515; 0.2105]      23.0       23.0
USA(UNC)    0.1783 [-0.9100; 1.2666]       1.5        1.5

Number of studies combined: k = 4

                          RD            95%-CI     z p-value
Fixed effect model   -0.0149 [-0.1495; 0.1198] -0.22  0.8288
Random effects model -0.0149 [-0.1495; 0.1198] -0.22  0.8288

Quantifying heterogeneity:
tau^2 = 0; H = 1.00 [1.00; 1.00]; I^2 = 0.0% [0.0%; 0.0%]

Test of heterogeneity:
    Q d.f. p-value
 0.30    3  0.9601

Details on meta-analytical method:
- Inverse variance method
- DerSimonian-Laird estimator for tau^2
```

```
kable(cbind(study=shannon.sex$studlab,pval=shannon.sex$pval))
```

| study | pval |
| --- | --- |
| Bangladesh | 0.987599902164408 |
| Haiti | 0.9752390484969 |
| USA(CA\_FL) | 0.622739246734807 |
| USA(UNC) | 0.748126030769876 |

```
# Other indexes
chao1.sex <- metagen(Estimate.genderMale, `Std. Error.genderMale`, studlab=pop,data=subset(asum4,id=="chao1"),sm="RD", backtransf=FALSE)
observed_species.sex <- metagen(Estimate.genderMale, `Std. Error.genderMale`, studlab=pop,data=subset(asum4,id=="observed_species"),sm="RD", backtransf=FALSE)
pd_whole_tree.sex <- metagen(Estimate.genderMale, `Std. Error.genderMale`, studlab=pop,data=subset(asum4,id=="pd_whole_tree"),sm="RD", backtransf=FALSE)
#show random meta-analysis model results of all indexes
atab<-as.data.frame(cbind(estimate=c(shannon.sex$TE.random,chao1.sex$TE.random,observed_species.sex$TE.random,pd_whole_tree.sex$TE.random),
                          ll=c(shannon.sex$lower.random,chao1.sex$lower.random,observed_species.sex$lower.random,pd_whole_tree.sex$lower.random),
                          ul=c(shannon.sex$upper.random,chao1.sex$upper.random,observed_species.sex$upper.random,pd_whole_tree.sex$upper.random),
                          index=c("shannon","chao1","observed_species","pd_whole_tree")))
atab[,1:3]<-lapply(atab[,1:3],as.character)
atab[,1:3]<-lapply(atab[,1:3],as.numeric)
a4<-ggplot(data=atab,aes(x=estimate,y=index))+
  geom_point(shape=16, colour="black")+
  geom_errorbarh(aes(xmin=ll,xmax=ul),height=0.0, colour="black")+
  geom_vline(xintercept=0,linetype="dashed")+
  scale_x_continuous(breaks=seq(from=-0.5,to=0.5,by=0.1),
                     labels=seq(from=-0.5,to=0.5,by=0.1))+
  theme(axis.line = element_line(colour = "black"),
        panel.grid.major = element_blank(),
        panel.grid.minor = element_blank(),
        panel.border = element_blank(),
        panel.background = element_blank())+
  xlab("Pooled standardized diversity difference")+ylab("Alpha diversity index")
a4
```

### Microbiome age

#### Predicting microbiome age, checking model performance, and replicate the results of the Bangladesh study

Random Forest (RF) modeling of gut microbiota maturity has been widely used to characterize development of the microbiome over chronological time. Adapting from the original approach of Subramanian et al, in the *microbiomeage* function, relative abundances of bacterial genera that were detected in the Bangladesh data and in the data of other studies to be included were regressed against infant chronological age using a RF model on a predefined training dataset of the Bangladesh study. This predefined training set includes 249 samples collected monthly from birth to 2 years of age from 11 Bangladeshi healthy singleton infants. The RF training model fit based on relative abundances of these shared bacterial genera was then used to predict infant age on the test data of the Bangladesh study and the data of each other study to be included. The predicted infant age based on relative abundances of these shared bacterial genera in each study is referred to as gut microbiota age.

In brief, the *microbiomeage* function get the shared genera list between the Bangladesh study and all other included studies, get the training and test sets from Bangladesh data based on the shared genera list, fit the train Random Forest model and predict microbiome age in the test set of Bangladesh data and data from all included studies, check for performance of the model based on the shared genera list on Bangladesh healthy cohort data, reproduce the findings of the Bangladesh malnutrition study.

The RF model based on the relative abundance of the shared bacterial genera of the four included studies explained 96% of the variance related to chronological age in the training set and 67% of the variance related to chronological age in the test set of Bangladesh data. This performance is better than the original RF model proposed by Subramanian et al.

```
#load Bangladesh taxa relative abundance summary up to genus level merged with mapping file (output from QIIME)
bal6<-read.delim(system.file("extdata/QIIME_outputs/Bangladesh/tax_mapping7", "Subramanian_et_al_mapping_file_L6.txt", package = "metamicrobiomeR", mustWork = TRUE))
colnames(bal6)<-tolower(colnames(bal6))
#View(bal6)
#format for data of other studies should be similar to Bangladesh data, must have 'age.sample' variable as age of infant at stool sample collection 
# Load data of 3 other studies 
data(gtab.3stud)
names(gtab.3stud)
```

```
[1] "nc"    "ca_fl" "haiti"
```

```
#predict microbiome age on Bangladesh data and data of other three studies based on shared genera across 4 studies  
#(take time to run)
#miage<-microbiomeage(l6.relabundtab=gtab.3stud)
#load saved results 
data(miage)
# list of shared genera that are available in the Bangladesh study and other included studies 
kable(miage$sharedgenera.importance)
```

| genera | importance |
| --- | --- |
| k\_\_bacteria.p\_\_firmicutes.c\_\_clostridia.o\_\_clostridiales.f\_\_lachnospiraceae.g\_\_blautia | 2481.8920619 |
| k\_\_bacteria.p\_\_firmicutes.c\_\_clostridia.o\_\_clostridiales.f\_\_ruminococcaceae.g\_\_ | 1906.7267503 |
| k\_\_bacteria.p\_\_firmicutes.c\_\_clostridia.o\_\_clostridiales.f\_\_lachnospiraceae.g\_\_ | 1382.4129745 |
| k\_\_bacteria.p\_\_bacteroidetes.c\_\_bacteroidia.o\_\_bacteroidales.f\_\_prevotellaceae.g\_\_prevotella | 650.0003357 |
| k\_\_bacteria.p\_\_firmicutes.c\_\_bacilli.o\_\_bacillales.f\_\_staphylococcaceae.g\_\_staphylococcus | 637.5007856 |
| k\_\_bacteria.p\_\_firmicutes.c\_\_clostridia.o**clostridiales.f**.g\_\_ | 632.9699303 |
| k\_\_bacteria.p\_\_firmicutes.c\_\_bacilli.o\_\_lactobacillales.f\_\_lactobacillaceae.g\_\_lactobacillus | 415.0586216 |
| k\_\_bacteria.p\_\_firmicutes.c\_\_clostridia.o\_\_clostridiales.f\_\_veillonellaceae.g\_\_dialister | 413.3290927 |
| k\_\_bacteria.p\_\_proteobacteria.c\_\_gammaproteobacteria.o\_\_pasteurellales.f\_\_pasteurellaceae.g\_\_haemophilus | 356.5366196 |
| k\_\_bacteria.p\_\_actinobacteria.c\_\_actinobacteria.o\_\_bifidobacteriales.f\_\_bifidobacteriaceae.g\_\_bifidobacterium | 312.9315215 |
| k\_\_bacteria.p\_\_actinobacteria.c\_\_actinobacteria.o\_\_actinomycetales.f\_\_actinomycetaceae.g\_\_actinomyces | 220.2466611 |
| k\_\_bacteria.p\_\_firmicutes.c\_\_clostridia.o\_\_clostridiales.f\_\_lachnospiraceae.g\_\_dorea | 202.6234334 |
| k\_\_bacteria.p\_\_firmicutes.c\_\_clostridia.o\_\_clostridiales.f\_\_lachnospiraceae.g\_\_.ruminococcus. | 158.0250246 |
| k\_\_bacteria.p\_\_firmicutes.c\_\_clostridia.o\_\_clostridiales.f\_\_lachnospiraceae.g\_\_coprococcus | 152.5757667 |
| k\_\_bacteria.p\_\_firmicutes.c\_\_bacilli.o\_\_lactobacillales.f\_\_enterococcaceae.g\_\_enterococcus | 147.6969771 |
| k\_\_bacteria.p\_\_proteobacteria.c\_\_gammaproteobacteria.o\_\_enterobacteriales.f\_\_enterobacteriaceae.g\_\_ | 134.5554227 |
| k\_\_bacteria.p\_\_firmicutes.c\_\_clostridia.o**clostridiales.f**.tissierellaceae..g\_\_anaerococcus | 134.5155377 |
| k\_\_bacteria.p\_\_firmicutes.c\_\_bacilli.o\_\_lactobacillales.f\_\_streptococcaceae.g\_\_streptococcus | 122.4810748 |
| k\_\_bacteria.p\_\_actinobacteria.c\_\_coriobacteriia.o\_\_coriobacteriales.f\_\_coriobacteriaceae.g\_\_collinsella | 117.5630377 |
| k\_\_bacteria.p\_\_firmicutes.c\_\_clostridia.o\_\_clostridiales.f\_\_veillonellaceae.g\_\_veillonella | 113.8576646 |
| k\_\_bacteria.p\_\_actinobacteria.c\_\_actinobacteria.o\_\_actinomycetales.f\_\_corynebacteriaceae.g\_\_corynebacterium | 107.8244500 |
| k\_\_bacteria.p\_\_firmicutes.c\_\_clostridia.o\_\_clostridiales.f\_\_clostridiaceae.g\_\_clostridium | 103.7414037 |
| k\_\_bacteria.p\_\_firmicutes.c\_\_erysipelotrichi.o\_\_erysipelotrichales.f\_\_erysipelotrichaceae.g\_\_ | 91.3499859 |
| k\_\_bacteria.p\_\_bacteroidetes.c\_\_bacteroidia.o\_\_bacteroidales.f\_\_bacteroidaceae.g\_\_bacteroides | 91.0539556 |
| k\_\_bacteria.p\_\_proteobacteria.c\_\_gammaproteobacteria.o\_\_pseudomonadales.f\_\_pseudomonadaceae.g\_\_pseudomonas | 89.6867279 |
| k\_\_bacteria.p\_\_actinobacteria.c\_\_actinobacteria.o\_\_actinomycetales.f\_\_micrococcaceae.g\_\_rothia | 73.2330650 |
| k\_\_bacteria.p\_\_firmicutes.c\_\_clostridia.o\_\_clostridiales.f\_\_clostridiaceae.g\_\_ | 73.1392380 |
| k\_\_bacteria.p\_\_actinobacteria.c\_\_coriobacteriia.o\_\_coriobacteriales.f\_\_coriobacteriaceae.g\_\_ | 70.4576219 |
| k\_\_bacteria.p\_\_proteobacteria.c\_\_betaproteobacteria.o\_\_neisseriales.f\_\_neisseriaceae.g\_\_neisseria | 63.0585978 |
| k\_\_bacteria.p\_\_firmicutes.c\_\_clostridia.o\_\_clostridiales.f\_\_ruminococcaceae.g\_\_oscillospira | 62.8580003 |
| k\_\_bacteria.p\_\_firmicutes.c\_\_erysipelotrichi.o\_\_erysipelotrichales.f\_\_erysipelotrichaceae.g\_\_.eubacterium. | 61.6988728 |
| k\_\_bacteria.p\_\_firmicutes.c\_\_bacilli.o\_\_gemellales.f\_\_gemellaceae.g\_\_ | 46.9889764 |
| k\_\_bacteria.p\_\_actinobacteria.c\_\_coriobacteriia.o\_\_coriobacteriales.f\_\_coriobacteriaceae.g\_\_atopobium | 46.8921466 |
| k\_\_bacteria.p\_\_fusobacteria.c\_\_fusobacteriia.o\_\_fusobacteriales.f\_\_fusobacteriaceae.g\_\_fusobacterium | 46.6647271 |
| k\_\_bacteria.p\_\_firmicutes.c\_\_clostridia.o**clostridiales.f**.tissierellaceae..g\_\_peptoniphilus | 45.0260938 |
| k\_\_bacteria.p\_\_proteobacteria.c\_\_betaproteobacteria.o\_\_burkholderiales.f\_\_alcaligenaceae.g\_\_sutterella | 38.8518496 |
| k\_\_bacteria.p\_\_firmicutes.c\_\_clostridia.o**clostridiales.f**.tissierellaceae..g\_\_finegoldia | 33.3776433 |
| k\_\_bacteria.p\_\_firmicutes.c\_\_bacilli.o\_\_lactobacillales.f\_\_streptococcaceae.g\_\_lactococcus | 32.9668029 |
| k\_\_bacteria.p\_\_firmicutes.c\_\_bacilli.o\_\_bacillales.f\_\_bacillaceae.g\_\_bacillus | 30.7285110 |
| k\_\_bacteria.p\_\_bacteroidetes.c\_\_bacteroidia.o\_\_bacteroidales.f\_\_porphyromonadaceae.g\_\_parabacteroides | 29.7347955 |
| k\_\_bacteria.p\_\_bacteroidetes.c\_\_bacteroidia.o\_\_bacteroidales.f\_\_rikenellaceae.g\_\_ | 24.7278422 |
| k\_\_bacteria.p\_\_firmicutes.c\_\_erysipelotrichi.o\_\_erysipelotrichales.f\_\_erysipelotrichaceae.g\_\_bulleidia | 7.2099213 |
| k\_\_bacteria.p\_\_firmicutes.c\_\_clostridia.o\_\_clostridiales.f\_\_veillonellaceae.g\_\_acidaminococcus | 6.5118551 |
| k\_\_bacteria.p\_\_cyanobacteria.c\_\_chloroplast.o**streptophyta.f**.g\_\_ | 6.1609772 |
| k\_\_bacteria.p\_\_firmicutes.c\_\_bacilli.o\_\_lactobacillales.f\_\_carnobacteriaceae.g\_\_granulicatella | 3.6444079 |
| k\_\_bacteria.p\_\_firmicutes.c\_\_clostridia.o\_\_clostridiales.f\_\_lachnospiraceae.g\_\_roseburia | 3.2288045 |
| k\_\_bacteria.p\_\_bacteroidetes.c\_\_flavobacteriia.o**flavobacteriales.f**.weeksellaceae..g\_\_cloacibacterium | 1.7980170 |
| k\_\_bacteria.p\_\_proteobacteria.c\_\_alphaproteobacteria.o\_\_rhizobiales.f\_\_rhizobiaceae.g\_\_agrobacterium | 1.4747628 |
| k\_\_bacteria.p\_\_firmicutes.c\_\_clostridia.o\_\_clostridiales.f\_\_ruminococcaceae.g\_\_anaerotruncus | 0.1673286 |
| k\_\_bacteria.p\_\_firmicutes.c\_\_bacilli.o\_\_bacillales.f\_\_paenibacillaceae.g\_\_paenibacillus | 0.1086412 |
| k\_\_bacteria.p\_\_proteobacteria.c\_\_alphaproteobacteria.o\_\_sphingomonadales.f\_\_sphingomonadaceae.g\_\_sphingomonas | 0.0342345 |
| k\_\_bacteria.p\_\_proteobacteria.c\_\_gammaproteobacteria.o\_\_pseudomonadales.f\_\_moraxellaceae.g\_\_ | 0.0106534 |

```
#check performance
grid.arrange(miage$performanceplot$ptrain, miage$performanceplot$ptest,nrow=1)
```

```
#replicate the findings of Subramanian et al paper 
ggplot() +geom_point(data=miage$microbiomeage.bangladesh$all,aes(x=age.sample, y=age.predicted, colour=health_analysis_groups))
```

#### Comparison of standardized predicted microbiome age between male vs. female infants <= 6 months of age adjusting for age of infants and breastfeeding status at sample collection using Bangladesh data

The predicted infant age in each included study based on relative abundance of the shared gut bacterial genera using the above RF model is referred to as gut microbiota age.Gut microbiotat age is standardized to have a mean of 0 and standard deviation of 1. Standardized gut microbiota age are compared between groups adjusting for covariates using LM and meta-analysis across studies is then done.

```
samhe<-merge(samde,he50[,c("child.id","gender","month.exbf","month.food")],by="child.id")
rmdat.rm<-merge(samhe,miage$microbiomeage.bangladesh$healthy,by.y="sampleid",by.x="fecal.sample.id")
# plot curves with GAMM 
rmdat.rm$gender<-as.factor(rmdat.rm$gender)
rmdat.rm$bf<-as.factor(rmdat.rm$bf)
gfit<-gamm(age.predicted~s(age.sample,by=gender) +gender,family=gaussian,
         data=rmdat.rm,random=list(personid=~1))
pred <- predict(gfit$gam, newdata = rmdat.rm,se.fit=TRUE)
datfit<-cbind(rmdat.rm, fit=pred$fit,ul=(pred$fit+(1.96*pred$se.fit)),ll=(pred$fit-(1.96*pred$se.fit)))
ggplot()+ geom_point(data = subset(rmdat.rm,age.sample<=6), aes(x = age.sample, y = age.predicted, group = personid, colour=gender),size=1)+ 
  geom_line(data = subset(rmdat.rm,age.sample<=6), aes(x = age.sample, y = age.predicted, group = personid, colour=gender),size=0.1)+
  geom_line(data = subset(datfit,age.sample<=6),aes(x = age.sample, y = fit, colour=gender),size = 1)+
  geom_ribbon(data = subset(datfit,age.sample<=6),aes(x=age.sample, ymax=ul, ymin=ll, fill=gender), alpha=.5)+guides(fill=FALSE)+
  xlab("Chronological age (month)") +ylab("Microbiome age (month)")+
  scale_x_continuous(breaks=seq(from=0,to=24,by=3),
                     labels=seq(from=0,to=24,by=3))+
  labs(color='')+
  theme(legend.position = "right",
        axis.line = element_line(colour = "black"),
        panel.grid.major = element_blank(),
        panel.grid.minor = element_blank(),
        panel.border = element_blank(),
        panel.background = element_blank(),
        strip.background =element_rect(fill="white"))
```

```
rmdat.rm$personid<-paste("rm",as.factor(tolower(rmdat.rm$personid)),sep=".")
rmdat.rm$sampleid<-paste("rm",tolower(rmdat.rm$fecal.sample.id),sep=".")
rmdat.rm$author<-"Subramanian et al"
rmdat.rm$pop<-"Bangladesh"
rmdat.rm$year<-"2014"
# standardize age.predicted to have mean of zero and standard deviation of 1 
rmdat.rm$age.predicteds<-(rmdat.rm$age.predicted-mean(rmdat.rm$age.predicted,na.rm=T))/sd(rmdat.rm$age.predicted)
# Comparison in infants <=6 months of age 
fitsum<-summary(lmer(age.predicteds~gender+bf+age.sample+(1|personid),data=subset(rmdat.rm,age.sample<=6)))
fitdat<-as.data.frame(fitsum$coefficients[-1,])
fitdat[,"varname"]<-rownames(fitdat)
fitdat[,"pop"]<-"Bangladesh"
kable(fitdat)
```

|  | Estimate | Std. Error | df | t value | Pr(>|t|) | varname | pop |
| --- | --- | --- | --- | --- | --- | --- | --- |
| genderMale | -0.0909496 | 0.0595166 | 36.06456 | -1.5281378 | 0.1352025 | genderMale | Bangladesh |
| bfNo\_BF | -0.1075706 | 0.1666272 | 248.75546 | -0.6455766 | 0.5191486 | bfNo\_BF | Bangladesh |
| bfNon\_exclusiveBF | 0.0339266 | 0.0571267 | 202.03700 | 0.5938845 | 0.5532537 | bfNon\_exclusiveBF | Bangladesh |
| age.sample | 0.0862128 | 0.0136845 | 236.02555 | 6.3000537 | 0.0000000 | age.sample | Bangladesh |

```
rm.ba.sex<-reshape(fitdat, idvar="pop", timevar="varname", direction="wide")
kable(rm.ba.sex)
```

|  | pop | Estimate.genderMale | Std. Error.genderMale | df.genderMale | t value.genderMale | Pr(>|t|).genderMale | Estimate.bfNo\_BF | Std. Error.bfNo\_BF | df.bfNo\_BF | t value.bfNo\_BF | Pr(>|t|).bfNo\_BF | Estimate.bfNon\_exclusiveBF | Std. Error.bfNon\_exclusiveBF | df.bfNon\_exclusiveBF | t value.bfNon\_exclusiveBF | Pr(>|t|).bfNon\_exclusiveBF | Estimate.age.sample | Std. Error.age.sample | df.age.sample | t value.age.sample | Pr(>|t|).age.sample |
| --- | --- | --- | --- | --- | --- | --- | --- | --- | --- | --- | --- | --- | --- | --- | --- | --- | --- | --- | --- | --- | --- |
| genderMale | Bangladesh | -0.0909496 | 0.0595166 | 36.06456 | -1.528138 | 0.1352025 | -0.1075706 | 0.1666272 | 248.7555 | -0.6455766 | 0.5191486 | 0.0339266 | 0.0571267 | 202.037 | 0.5938845 | 0.5532537 | 0.0862128 | 0.0136845 | 236.0255 | 6.300054 | 0 |

The analyses for other studies were done similarly.

#### Meta-analysis of four studies

The results showed that standardized microbiota age was significantly different between males vs. females but in opposite directions in two studies with small sample sizes (Haiti and North Carolina). However, meta-analysis of all four studies revealed no significant difference in gut microbiota age between genders after adjusting for feeding status and infant age at time of sample collection.

```
#load saved results of four studies
data(rm4.sexs)
kable(rm4.sexs)
```

| pop | Estimate.genderMale | Std. Error.genderMale | df.genderMale | t value.genderMale | Pr(>|t|).genderMale | Estimate.bfNon\_exclusiveBF | Std. Error.bfNon\_exclusiveBF | df.bfNon\_exclusiveBF | t value.bfNon\_exclusiveBF | Pr(>|t|).bfNon\_exclusiveBF | Estimate.bfNo\_BF | Std. Error.bfNo\_BF | df.bfNo\_BF | t value.bfNo\_BF | Pr(>|t|).bfNo\_BF | Estimate.age.sample | Std. Error.age.sample | df.age.sample | t value.age.sample | Pr(>|t|).age.sample |
| --- | --- | --- | --- | --- | --- | --- | --- | --- | --- | --- | --- | --- | --- | --- | --- | --- | --- | --- | --- | --- |
| Bangladesh | -0.0982571 | 0.0635555 | 36.765259 | -1.5460054 | 0.1306687 | 0.0466096 | 0.0598532 | 207.991061 | 0.7787320 | 0.4370226 | -0.0752122 | 0.1738326 | 248.95022 | -0.432670 | 0.6656291 | 0.0895102 | 0.0142403 | 235.73826 | 6.285695 | 0.0000000 |
| Haiti | 0.5595183 | 0.2722964 | NA | 2.0548132 | 0.0461522 | -0.3112344 | 0.3232793 | NA | -0.9627416 | 0.3411877 | NA | NA | NA | NA | NA | 0.2466495 | 0.0802007 | NA | 3.075402 | 0.0036890 |
| USA(CA\_FL) | -0.1007744 | 0.1133121 | 69.802832 | -0.8893527 | 0.3768683 | 0.2476086 | 0.1134385 | 148.116702 | 2.1827570 | 0.0306289 | 0.9031475 | 0.2439538 | 171.39469 | 3.702126 | 0.0002880 | 0.2696717 | 0.0282094 | 205.87222 | 9.559643 | 0.0000000 |
| USA(NC) | -0.7286074 | 0.3616621 | 3.088779 | -2.0146081 | 0.1347374 | 1.0076149 | 0.3132025 | 5.097796 | 3.2171356 | 0.0229018 | -0.8707151 | 0.6762228 | 11.38869 | -1.287616 | 0.2234301 | 0.4687383 | 0.0845192 | 15.95532 | 5.545941 | 0.0000447 |

```
rm.sex<-metagen(Estimate.genderMale, `Std. Error.genderMale`, studlab=pop,data=rm4.sexs,sm="RD", backtransf=FALSE)
forest(rm.sex,smlab="Standardized \n microbiome age difference",lwd=2)
```

```
rm.sex
```

```
                RD             95%-CI %W(fixed) %W(random)
Bangladesh -0.0983 [-0.2228;  0.0263]      71.4       40.7
Haiti       0.5595 [ 0.0258;  1.0932]       3.9       15.4
USA(CA_FL) -0.1008 [-0.3229;  0.1213]      22.5       33.7
USA(NC)    -0.7286 [-1.4375; -0.0198]       2.2       10.2

Number of studies combined: k = 4

                          RD            95%-CI     z p-value
Fixed effect model   -0.0871 [-0.1924; 0.0181] -1.62  0.1048
Random effects model -0.0625 [-0.3203; 0.1953] -0.48  0.6348

Quantifying heterogeneity:
tau^2 = 0.0385; H = 1.72 [1.00; 2.94]; I^2 = 66.0% [0.4%; 88.4%]

Test of heterogeneity:
    Q d.f. p-value
 8.83    3  0.0316

Details on meta-analytical method:
- Inverse variance method
- DerSimonian-Laird estimator for tau^2
```

```
kable(cbind(study=rm.sex$studlab,pval=rm.sex$pval))
```

| study | pval |
| --- | --- |
| Bangladesh | 0.122103269327307 |
| Haiti | 0.0398970499413607 |
| USA(CA\_FL) | 0.373813543114747 |
| USA(NC) | 0.0439457250978759 |

# Discussion and Conclusion

Our *metamicrobiomeR* package implemented GAMLSS-BEZI for analysis of microbiome relative abundance data and random effect meta-analysis models for meta-analysis across microbiome studies. The advantages of GAMLSS-BEZI are: 1) it directly and properly address the distribution of microbiome relative abundance data which resemble a zero-inflated beta distribution; 2) it has better power to detect differential relative abundances between groups than the commonly used approach LMAS; 3) the estimates from GAMLSS-BEZI are log(odds ratio) of relative abundances between groups and thus are comparable across studies. Random effects meta-analysis models can be directly applied to pool these adjusted estimates and their standard errors across studies. This approach allows examination of study-specific effects, heterogeneity between studies, and the overall pooled effects across microbiome studies. Besides, random effects meta-analysis models can also generally applied to other microbiome measures such as diversity indexes or microbiome age. Standardization of these measures before comparison between groups within each study also make the estimates for these measures comparable across studies. The examples and workflow using our *metamicrobiomeR* package are reproducible and applicable for the analysis and meta-analysis of other microbiome studies.

# Availability

All source code, example data, documentation and the manuscript describing the *metamicrobiomeR* package are available at [https://github.com/nhanhocu/metamicrobiomeR].

# Funding

This work was supported by Mervyn W. Susser fellowship in the Gertrude H. Sergievsky Center, Columbia University Medical Center (to Nhan Thi Ho) during the development and supported by Vinmec Healthcare System, Vietnam (to Nhan Thi Ho) during the revision.

# Section information

```
sessionInfo()
```

```
R version 3.5.2 (2018-12-20)
Platform: x86_64-w64-mingw32/x64 (64-bit)
Running under: Windows 10 x64 (build 17134)

Matrix products: default

locale:
[1] LC_COLLATE=English_United States.1252 
[2] LC_CTYPE=English_United States.1252   
[3] LC_MONETARY=English_United States.1252
[4] LC_NUMERIC=C                          
[5] LC_TIME=English_United States.1252    

attached base packages:
[1] stats     graphics  grDevices utils     datasets  methods   base     

other attached packages:
 [1] meta_4.9-4          mgcv_1.8-27         nlme_3.1-137       
 [4] lmerTest_3.1-0      lme4_1.1-20         Matrix_1.2-15      
 [7] ggplot2_3.1.0       gridExtra_2.3       gdata_2.18.0       
[10] dplyr_0.8.0.1       plyr_1.8.4          metamicrobiomeR_1.1
[13] usethis_1.4.0       devtools_2.0.1      knitr_1.21         

loaded via a namespace (and not attached):
 [1] pkgload_1.0.2      splines_3.5.2      foreach_1.4.4     
 [4] prodlim_2018.04.18 gtools_3.8.1       assertthat_0.2.0  
 [7] stats4_3.5.2       highr_0.7          yaml_2.2.0        
[10] remotes_2.0.2      ipred_0.9-8        sessioninfo_1.1.1 
[13] numDeriv_2016.8-1  pillar_1.3.1       backports_1.1.3   
[16] lattice_0.20-38    glue_1.3.0         digest_0.6.18     
[19] RColorBrewer_1.1-2 minqa_1.2.4        colorspace_1.4-0  
[22] recipes_0.1.4      htmltools_0.3.6    timeDate_3043.102 
[25] pkgconfig_2.0.2    caret_6.0-81       purrr_0.3.0       
[28] scales_1.0.0       processx_3.2.1     gower_0.1.2       
[31] lava_1.6.5         tibble_2.0.1       generics_0.0.2    
[34] withr_2.1.2        nnet_7.3-12        lazyeval_0.2.1    
[37] cli_1.0.1          survival_2.43-3    magrittr_1.5      
[40] crayon_1.3.4       memoise_1.1.0      evaluate_0.13     
[43] ps_1.3.0           fs_1.2.6           MASS_7.3-51.1     
[46] class_7.3-14       pkgbuild_1.0.2     tools_3.5.2       
[49] data.table_1.12.0  prettyunits_1.0.2  stringr_1.4.0     
[52] munsell_0.5.0      callr_3.1.1        compiler_3.5.2    
[55] rlang_0.3.1        grid_3.5.2         nloptr_1.2.1      
[58] iterators_1.0.10   labeling_0.3       rmarkdown_1.11    
[61] gtable_0.2.0       ModelMetrics_1.2.2 codetools_0.2-15  
[64] curl_3.3           reshape2_1.4.3     R6_2.4.0          
[67] lubridate_1.7.4    rprojroot_1.3-2    desc_1.2.0        
[70] stringi_1.3.1      Rcpp_1.0.0         rpart_4.1-13      
[73] tidyselect_0.2.5   xfun_0.5
```

# References

---

1. Rigby RA, Stasinopoulos DM. Generalized additive models for location, scale and shape (with discussion). J R Stat Soc Ser C (Applied Stat). 2005;54:507-54.↩
2. Subramanian S, Huq S, Yatsunenko T, Haque R, Mahfuz M, Alam MA, et al. Persistent gut microbiota immaturity in malnourished Bangladeshi children. Nature. 2014;510:417-21.↩
3. Bender JM, Li F, Martelly S, Byrt E, Rouzier V, Leo M, et al. Maternal HIV infection influences the microbiome of HIV-uninfected infants. Sci Transl Med. 2016;8:349ra100.↩
4. Pannaraj PS, Li F, Cerini C, Bender JM, Yang S, Rollie A, et al. Association Between Breast Milk Bacterial Communities and Establishment and Development of the Infant Gut Microbiome. JAMA Pediatr. 2017;90095:647-54.↩
5. Thompson AL, Monteagudo-Mera A, Cadenas MB, Lampl ML, Azcarate-Peril MA. Milk- and solid-feeding practices and daycare attendance are associated with differences in bacterial diversity, predominant communities, and metabolic and immune function of the infant gut microbiome. Front Cell Infect Microbiol. 2015;5:3.↩
